# Inheritance and QTL Mapping of Leaf Nutrient Concentration in a Cotton Inter-Specific Derived RIL Population

**DOI:** 10.1371/journal.pone.0128100

**Published:** 2015-05-28

**Authors:** Shiming Liu, Jean-Marc Lacape, Greg A. Constable, Danny J. Llewellyn

**Affiliations:** 1 CSIRO Agriculture Flagship, Narrabri, NSW 2390, Australia; 2 CIRAD, UMR-AGAP, Avenue Agropolis, F-34398, Montpellier, France; 3 CSIRO Agriculture Flagship, P.O. Box 1600, Canberra, ACT 2601, Australia; Department of Agriculture and Food Western Australia, AUSTRALIA

## Abstract

Developing and deploying cotton cultivars with high nutrient uptake, use efficiency and tolerance to nutrient related soil stresses is desirable to assist sustainable soil management. Genetic variation, heritability, selection response and quantitative trait loci (QTLs) were investigated for five macronutrients (P, K, Ca, Mg, S) and five micronutrients (Fe, Mn, B, Zn, and Cu) in a recombinant inbred line (RIL) population from an inter-specific cross between *Gossypium hirsutum* cv. Guazuncho 2, and *G*. *barbadense* accession VH8-4602. Na and K/Na ratio were also studied as the imbalance between Na and other nutrients is detrimental to cotton growth and development. The concentrations of nutrients were measured for different plant parts of the two parents and for leaf samples of the whole population collected at early to peak flowering in field experiments over two years in a sodic Vertosol soil. Parental contrast was large for most nutrient concentrations in leaves when compared with other plant parts. Segregation for leaf nutrient concentration was observed within the population with transgression for P, K, K/Na ratio and all micronutrients. Genotypic difference was the major factor behind within-population variation for most nutrients, while narrow sense heritability was moderate (0.27 for Mn and Cu, and 0.43 for B). At least one significant QTL was identified for each nutrient except K and more than half of those QTLs were clustered on chromosomes 14, 18 and 22. Selection response was predicted to be low for P and all micronutrients except B, high for K, Na and B, and very high for K/Na ratio. Correlations were more common between macronutrients, Na and K/Na ratio where the nature and strength of the relations varied (r=-0.69 to 0.76). We conclude that there is sufficient genetic diversity between these two tetraploid cotton species that could be exploited to improve cotton nutrient status by introgressing species-unique favourable alleles.

## Introduction

Mineral nutrients are important for the biochemical and biophysical processes responsible for plant growth and development [1]. Cotton is a significant field crop produced for fibre as well as oil around the world and takes up at least six macro- and nine micro-nutrients in its life cycle [2]. To improve economic viability, a cotton producer requires higher yields, higher prices or lower costs. Higher yield may require better crop nutrition with greater uptake of essential or limiting nutrients [3]. However, excessive fertilser input in cropping can cause both economic and environmental concerns. On Australian cotton farms, for example, the agronomic cost of fertilsers doubled between 2003 and 2012 due to both higher prices and increased fertilser application [4]. The economic reward from such practices will progressively become diminished unless yields can keep pace through positive interactions between new cultivars and agronomic innovations [5]. Meanwhile, the use efficiency of fertilsers remains low in field crops [6] and fertilser application has been responsible for the increased nutrient rich runoff detrimental to natural waterways [6], and increased salt deposition in soils [7].

Cotton is grown on a wide range of soils [7]. Soil associated nutrient constraints or deficiencies, imbalances, interactions or toxicities, which occur at various scales in different regions and production systems, can limit cotton yield and/or fibre quality [8–9]. N, P and K are used as fertilser on cotton worldwide [7, 10]. Their deficiency can reduce yield by up to 65% [11–12]. Cotton is reputed to be inefficient in taking up K [13], and K deficiency is often observed in soils with low K availability [13–14] and also in soil with sufficient K, but with a crop with a high yield [15]. A balanced supply of nutrients is required—the ratios of P and K with S are known to be important to optimum cotton growth and development [16]. Zn deficiency may occur with cotton in alkaline soils [17] and also after a long fallow, due to reduced soil mycorrhizae that assist root uptake of P and Zn especially [18]. To a lesser extent, nutrient toxicities can occur due to excessive soil micronutrients such as Mn, Zn, B and Cu [8–9]. Waterlogged soil conditions produce multiple nutritional problems, including Mn toxicity and Fe deficiency [10].

Soil salinity and/or sodicity are significant global constraints for irrigated cotton production from excess salt or sodium in the soil profile [19]. Despite Na not being commonly considered as a nutrient, it can partially substitute for K, Ca and Mg in cotton metabolic and physical processes when these nutrients are in short supply [20–22] and the imbalance of Na with other nutirents has detrimental effect on cotton growth and development. In sodic soils of Australia [23], Na can limit cotton uptake of P and K under some conditions including waterlogging [10] and high fruit load [15]. Sodium sequestration and exclusion are proposed as mechanisms behind salinity tolerance in many plant species and also cotton, so Na concentrations in leaf and shoot are often measured to assess plant nutrient status. Shoot concentrations of Na, K and Ca of cotton under saline conditions were proved to be governed by additive gene effects with heritability estimates from 0.34 to 0.51 [24]. In tomato and rice, moderate to high heritability for Na accumulation were reported under saline conditions [25–26]; while in wheat, a number of QTLs were identified for Na^+^ exclusion [27].

Soil associated nutrient constraints for cotton cannot always be mitigated completely by soil amelioration and/or fertilser application. There is interest in many crops to develop and deploy new genetic material to improve plant uptake and use efficiency of nutrients and also to overcome nutrient constraints [6, 28–29]. Compared with agronomic approaches, the genetic approach has been expected to deliver a cost effective and durable solution, but there has been limited research to identify genetic variation in cotton to improve cotton nutrient uptake, use efficiency and tolerance to nutrient constraints. Among the two cultivated tetraploid cotton species, *Gossypium barbadense (Gb)* was found more capable of K uptake [30] and inter-varietal differences within *G. hirsutum (Gh)* were reported for uptake and use efficiency of P [31] and K [29, 32]. In two cultivars with high and low K use efficiency, yield response to K suggested that the K-use efficient cultivar required a lower critical leaf and soil K content [29]. *Gb* has been found to have better tolerance than *Gh* to soil salinity and sodicity [33]. Either straight crossing or backcrossing was effective in transferring high salt tolerance from *Gb* to *Gh* [34]. There was a range of salinity tolerance among *Gh* lines [28]. Genetic difference in tolerance to micronutrient imbalance, such as Mn, has been reported in *Gh* [35–36]. Most recently, both nutrient uptake and use efficiency were found increased concurrently with yield progress of cotton cultivars released from 1973 to 2006 in Australia, although the highest rate of increase was lint yield [37].

Evidence for cotton responses to nutrient uptake, utilisation and constraints has usually been obtained under imposed nutrient stress conditions. The importance of stress conditions or treatments cannot be underestimated as a powerful screen to differentiate plant responses and to identify desired germplasm. On the other hand, in plant salt tolerance QTL mapping, both constitutive and induced genetic QTLs were found responsible for salt tolerance in tomato [38] and wheat [27], and the constitutive QTLs exhibited large individual effects and contributed to a greater proportion of the total phenotypic variation [38]. If this is applicable to other plant nutrients, the constitutive genetic components for the nutrients can be understood under non-stress conditions based on phenotyping data of a segregating genetic population, as has been done for yield components and fibre quality with the same population reported here [39–40]. Genetic understanding for Fe and Zn concentrations in cereals and beans are examples of such research focussed on developing new cultivars with micronutrient rich grains useful to overcome human nutritional deficiencies [41]. Furthermore, in seeds of various species, including Arabidopsis [42], Brassica [43], rice [44], wheat [45] or Medicago [46], genetic mapping populations have been developed to identify QTLs that contribute to mineral concentrations. However, the genetic basis for the processes of mineral nutrient uptake, translocation and accumulation in plants is essentially unknown.

In this study, five macro-, five micro-nutrient and Na concentrations in leaves were measured on 77 lines in a cotton recombinant inbred line (RIL) population from an inter-specific cross of *Gb* and *Gh* grown in non-nutrient stress experiments over two seasons on a typical Australian sodic alkaline clay Vertosol. Quantitative genetics and quantitative trait loci (QTLs) mapping were applied in data analysis to reveal their phenotypic variability, heritability, predicted selection response and interrelationships. The information from this study is aimed at identifying traits and so developing breeding strategies to improve cotton nutrient uptake, use efficiency and tolerance to nutrient related stresses.

## Materials and Methods

### RIL population and field experiments

The RIL population was derived from an inter-specific cross between the Argentinean *Gh* cv. Guazuncho 2 (GUA) and the *Gb* accession VH8-4602 (VH8) [47]. The field experiments consisted of 93 and 82 lines in 2007/08 and 2008/09, respectively. They were at F_7_ generation or later. Eleven RILs were excluded from the 2008/09 experiment, as like the *Gb* parent VH8, they were vegetatively vigorous and late maturing under Australian conditions and did not produce enough seeds in 2007/08 to allow planting in 2008/09. Two locally bred controls, Sicot 75 (*Gh*) and Sipima 280 (*Gb*) were included in the experiments. The experiments were arranged as a partial replicated design with the detail and planting row-spacing as described previously [40].

The experiments in both seasons were carried out in the CSIRO Leitch lease (E 30° 10’, S149° 35’) near Narrabri, NSW, Australia, on a self mulching Vertosol classified as a fine, thermic, montmorillonitic Typic Haplustert [48]. The soil originated from native grasses and woodland and has a uniform profile of medium to heavy clay with the surface soil pH (1:5 soil:water) of 8.1 and 8.5 at 1 m depth [23]. The field was under a two-year cotton-wheat-fallow rotation system, and N fertilser as anhydrous ammonia was injected at 20 cm into the soil at a rate of 180 kg N/ha 6 weeks prior to sowing. Other nutrients were not limiting [10]. Cotton crops were furrow-irrigated regularly to avoid drought stress when soil water deficit approached 50 mm. Insects were controlled when they exceeded commercial thresholds [49].

### Plant samples for nutrient concentration analysis

In this study, leaf nutrient concentrations at early to peak flowering were used to assess phenotypic differences in nutrient accumulation of the RIL population. This is because the fully expanded young leaves in the upper part of the cotton plant are one of the main sinks for nutrients as well as a centre for photosynthesis. Peak nutrient uptake and accumulation in cotton occurs at early to peak flowering which is just after rapid growth and expansion of the root system and prior to the beginning of the maximal above ground growth [2, 50–51]. Through that period, cotton plants can take up nutrients at their highest rate and accumulate about 50% or more of the nutrient amounts required for the entire crop [2–3]. After that, nutrients can be translocated from the leaves and utilised to meet the needs of ongoing vegetative and particularly reproductive growth; and plant nutrient uptake and accumulation slow during boll development and maturation [2]. Therefore, phenotypic variability in leaf nutrient concentrations at the early to peak flowering stage should represent the underlying genetic capacities for nutrient uptake, accumulation and utilisation, which are fundamental processes associated with plant nutrient use efficiency [52].

In the experiments, 10 leaves were randomly collected from each plot at early to peak flowering (about 88 days after planting in both years). The leaves were young, fully expanded and undamaged at the fourth node beneath the plant terminal. The samples were dried in a forced draught dehydrator at 70°C for 48 hours and then finely milled.

In 2008/09 in order to measure nutrient concentrations of all plant parts, three whole plants at peak flowering were dug up from each of three replicated plots of the parental lines in the experiment. After roots were cleaned with rain water, plants were divided into leaf, petiole, stem, seed and root, and dried in a dehydrator and finely milled.

The concentrations of P, K, Ca, Mg, S, Na, Fe, Mn, B, Cu, and Zn for all plant samples were determined by Inductively Coupled Plasma Atomic Emission Spectrometry (ICPAES) after digestion in nitric and perchloric acids [53]. K/Na ratio was calculated based on leaf K and Na concentration to determine phenotypic difference in Na exclusion and/ or substitution of K.

### Data analysis

Analysis of variance was conducted for each nutrient of the tissue samples from the two parental lines. The analysis was processed by treating plot as block factor and line and plant part as treatment factors. Mean estimates for each plant part of the parental lines were compared using least squared mean difference at *P*<0.05 (lsd_.05_), due to statistical significance of line × plant part interactions for all nutrient variables except for B (F value> 2.89 and < 5175.6, P_(4, 29)_ ≤ 0.05).

A combined analysis was conducted for each nutrient concentration of leaf samples of the RIL population over the two seasons. In the analysis, season was fitted as fixed and test line and its interaction with season as random. According to the experiment design, local plot error was fitted with a two dimensional spatial model using the first order separable autoregressive (AR1) variance-covariance structure; other spatial variations, namely, global trend and extraneous variation along with experiment dimensions, were added in the model when required according to a Wald test and sample variogram [54], because such variations are common in cotton breeding experiments under irrigated conditions [55]. The repeat plot effect of some RILs in the experiment was counted by fitting an extra random factor in the model. Finally, empirical best linear unbiased predictions (E-BLUPs) were obtained for 77 RILs, parents and controls tested in common in both seasons.

Variance estimates for the population (V_RIL_) was obtained for each nutrient when the final model used in the above analysis was re-fitted by adding a new fixed term which treated RILs, the two parents and controls as different levels. Heritability in the narrow sense (h^2^) was calculated using the formula of 100*0.5*V_RIL_/V_P,_ where Vp represents phenotypic variance and is the sum of variance components of genotype (V_g_), genotype × season interaction (V_g × s_) and the error variance (V_error_), under the assumption of non-allelic interactions between different loci (i.e. epistasis) for a quantitative trait in a RIL population from a bi-parent cross [56]. Standard error of heritability estimates were derived based on the delta method using REML estimated variance-covariance matrix [57]. The selection response was predicted for the scenarios in which the top 5% and 10% of RILs in the target direction were retained using the equation of R = i* √V_p_ *h^2^, where i is the standardised selection differential i.e. selection intensity, and can be estimated when trait phenotypic variation follows a normal distribution in a breeding population [58].

D’Agostino skewness and Anscombe-Glynn kurtosis tests were used to detect any departure from a normal distribution for each nutrient in the RIL population. Pearson’s correlation analysis was conducted between nutrient variables using E-BLUPs of the RIL population and between nutrient variables, yield and fibre properties, the latter two were already reported in our previous paper [40]. The E-BLUPs of individual nutrients were strongly correlated between the two-year experiments (r ranged from 0.60 for Mn to 0.95 for Zn, P<0.001)(S1 Table). The above analysis was carried out using ASReml-R [59] and R software (http://www.r-project.org/).

### QTL analysis

**Fig 1:**
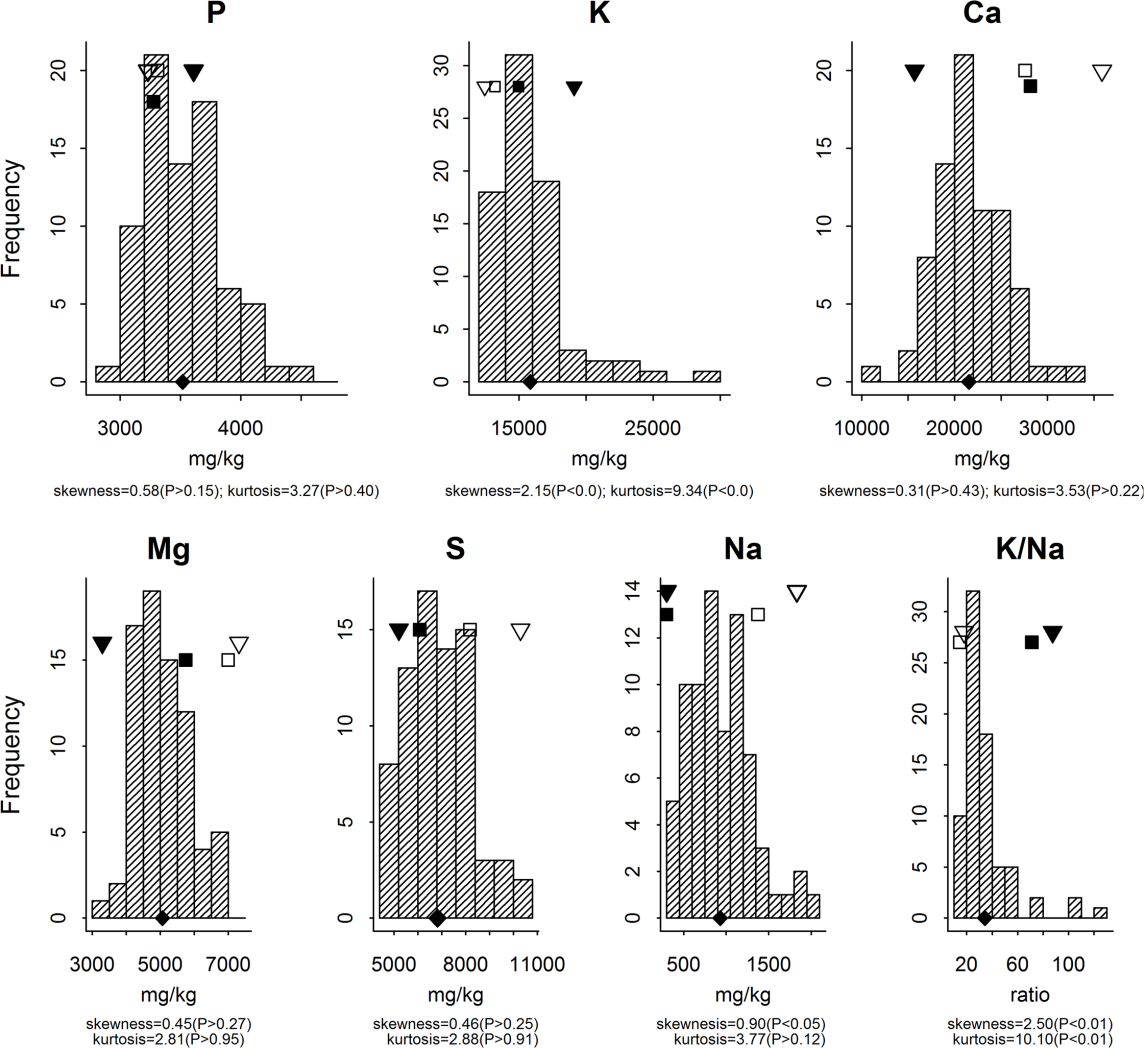
Distribution and normality tests of five macronutrient and Na concentrations and K/Na ratio in leaves of 77 RILs. ▼ = VH8; **▽** = GUA; ■ = Sipima 280, □ = Sicot 75; ◆ = population mean.

QTL analysis was performed as described previously [39]. In brief, a subset of 656 loci evenly distributed on the RIL map [47] served for interval mapping (IM) and composite interval mapping (CIM, model 6) using WinQTL Cartographer 2.5 [60]. The predictions from the combined analysis were used for QTL discovery and mapping. Among the 12 (E-BLUPs) variables, K and K/Na ratio deviated from normality and were log-transformed (Fig 1), the 10 others (P, Ca, Mg, Na, S, Fe, Mn, B, Cu, and Zn) were not transformed. A total of 74 RILs were analysed for QTLs after the other three shown as off-types (>98% GUA) and were discarded. For each variable, the minimum significant LOD (global risk of 5%) threshold score was determined after 1000 permutations. The results of the QTL position, their 1LOD or 2LOD drop-off confidence intervals, proportion of phenotypic variance explained (R²), and additive effect reported are those derived from CIM. Chromosome numbering followed the classical nomenclature system: c1-c13 and c14-c26 for the chromosomes of the A_t_ and D_t_ sub-genomes, respectively. Those significant QTLs with LOD above the permutation threshold for each trait were supplemented by putative QTLs with LOD>2.5, that were used to infer co-localizations of QTLs for different traits. The permutation-based LOD thresholds varied between 3.34 for S and 3.91 for log K. Graphical representations were generated with the MapChart software [61]. Cases of co-localizations of QTLs were inferred by their overlapping confidence intervals.

## Results

### Phenotypic differences of leaf nutrient concentration between parents and within the RIL population

**Fig 2:**
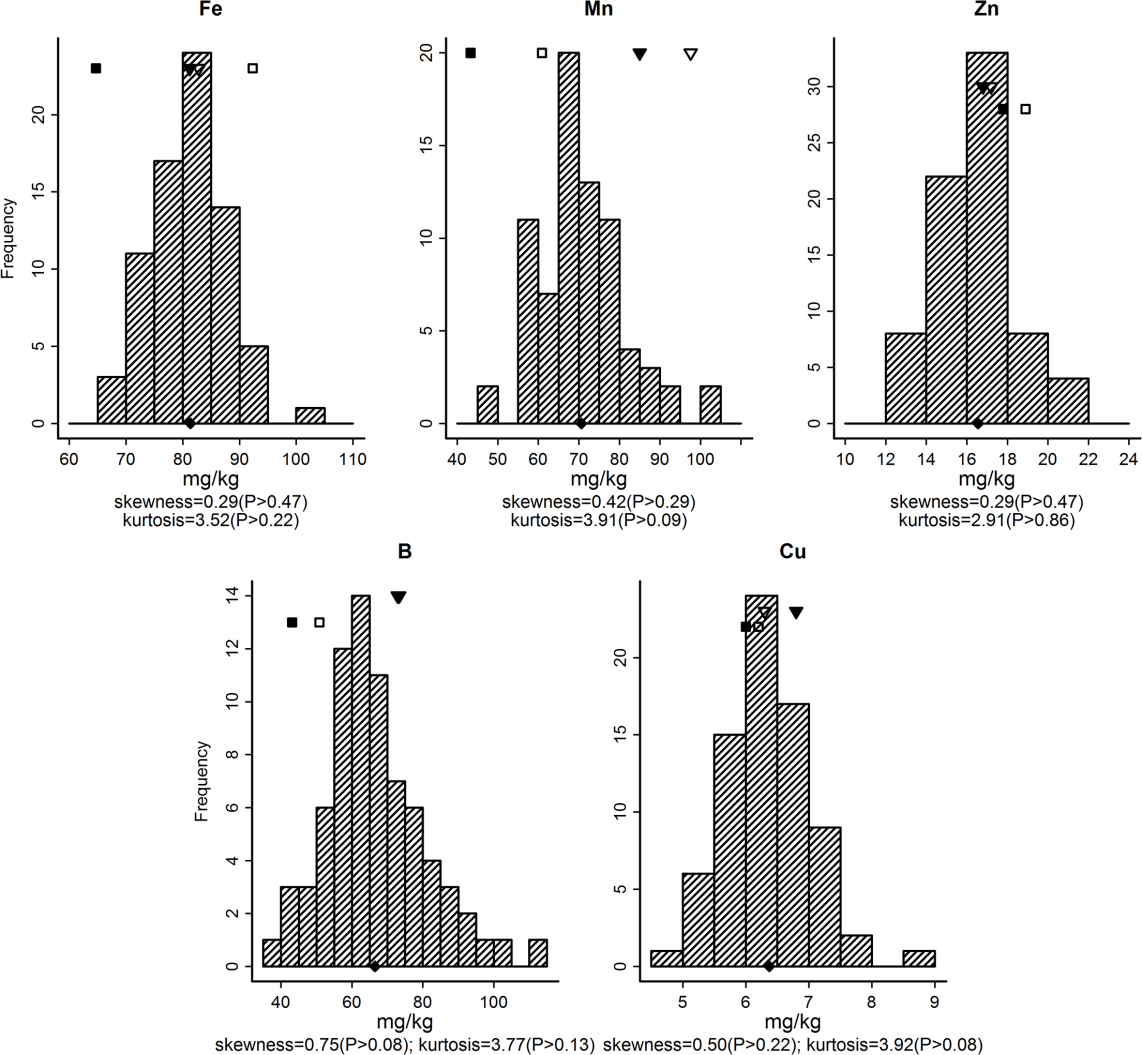
Distribution and normality tests of five micronutrient concentration in leaves of 77 RILs. ▼ = VH8; **▽** = GUA; ■ = Sipima 280, □ = Sicot 75; ◆ = population mean.

Nutrient concentration varied with parents and plant parts except for B, for which the line × plant part interaction was not significant (Table 1). Nutrient concentration was the highest in leaves or petioles except for P, Na, Fe, Zn and Cu, whereas seeds had the highest concentration for P, Zn and Cu, stem for Na and root for Fe. Also in leaves and petioles, parental contrast was more apparent and consistent. Based on leaf nutrient concentrations, the GUA parent had higher concentration for Ca, Mg, S, Na, and Mn, and VH8 was higher for P and K and also high K/Na ratio with no difference for micronutrients Fe, Zn, B and Cu. Similar species differences were observed between the local *Gh* and *Gb* controls for the nutrients mentioned above plus Fe (Figs 1 and 2), but the controls had low concentrations of Mn, B and Cu in leaves compared with parental lines of the RIL population that are not specifically adapted to Australian growing conditions.

**Table 1:** Nutrient, Na concentrations and K/Na ratio in different plant parts of two parents of the RIL population, Guazuncho 2 (GUA), *Gossypium hirsutum*, and VH8-4602 (VH8), *G*. *barbadense*.

Part	Parent	Concentration (mg/kg)											
		P	K	Ca	Mg	S	Na	K/Na	Fe	Mn	Zn	B	Cu
Leaf	GUA	**2300**	**10767**	**52333**	**8967**	**13767**	**3067**	**3.5**	169.0	**135.7**	15.4	82.8	7.1
	VH8	**3100**	**22000**	**25667**	**5067**	**6667**	**870**	**34.2**	119.0	**69.8**	16.9	86.1	7.7
Petiole	GUA	1463	**33333**	**22500**	**10400**	**3467**	**3233**	**10.4**	72.1	**38.4**	**7.0**	28.5	**4.4**
	VH8	1830	**39333**	**14233**	**6667**	**1413**	**1027**	**48.7**	55.0	**19.8**	**11.1**	27.1	**5.9**
Stem	GUA	**1147**	17800	11733	5200	**2667**	**3564**	4.6	85.0	**22.4**	**9.7**	21.3	12.8
	VH8	**2013**	19767	8233	3900	**1490**	**1210**	26.4	49.0	**14.6**	**17.5**	17.7	11.5
Root	GUA	**623**	9067	2867	1810	780	**953**	9.5	**333.0**	12.4	6.1	10.5	**3.7**
	VH8	**1067**	9667	4067	1960	667	**1970**	5.4	**522.0**	14.9	8.8	9.5	**5.3**
Seed	GUA	**5561**	11200	973	3067	2667	125	**92.7**	47.0	13.8	**21.6**	13.3	**8.2**
	VH8	**4167**	11867	1187	2467	1873	2	**2308.4**	76.0	10.7	**19.9**	11.3	**9.8**
lsd_0.05_		392.8	3714.5	3618.9	1127.3	821.6	621.5	28.9	126.1	3.6	2.4	6.1	1.5

The pairs of bold values within the same plant tissue differed significantly between parents at P≤0.05.

Distribution of macronutrient and Na concentrations and K/Na ratio in leaves for the RIL population are given in Fig 1. The variation of the RIL population followed a normal distribution for the concentration of P, Ca, Mg and S, but a distribution with positive skewness and/or kurtosis for K, Na and K/Na ratio. The population mean was towards the higher parent for P, mid-parent value for K, Mg and Na, but the lower parent for Ca, S and K/Na ratio. There were a few RILs with the concentration of P, K, S and K/Na ratio higher than the higher parent. When compared with the controls, more RILs exhibited higher than the higher control for the concentrations of P, K, Ca, S and K/Na ratio. However, no RILs had lower Na concentration than the lower parent or control.

Distribution of micronutrient concentrations in leaves of the RIL population are given in Fig 2. All micronutrients followed a normal distribution. The population mean was less than the lower parent for Mn, B and Cu, but close to mid-parent value for Fe and Zn. There was a large proportion (69% for Cu to 96% for B) of RILs outside the concentration range found in the two parents and controls.

### Heritability estimates and predicted selection response

Both genotype and genotype × season interaction contributed significantly to phenotypic variability of nutrients in the RIL population with the former being more important (Table 2). Large error variance estimates associated with the concentration of Fe, Cu and P suggested factors beyond those accounted by the experiment design and data analysis were an important source of their observed phenotypic variation.

**Table 2:** Importance of variation source for five macro-, five micro-nutrient and Na concentrations and K/Na ratio on leaves revealed from a combined data analysis over two seasons.

Nutrient	Source (%)		
	Genotype	Genotype × Season	Error
P	45.8	17.8	36.3
K	57.0	22.2	20.9
Ca	58.2	28.2	13.6
Mg	59.1	22.9	18.0
S	47.3	35.3	17.4
Na	46.6	27.9	25.5
K/Na	66.9	13.2	19.9
Fe	33.8	23.8	42.4
Mn	36.8	35.3	27.9
B	71.0	14.2	14.8
Cu	29.7	28.3	42.0
Zn	61.4	10.6	28.0

Heritability estimates and predicted selection response are given in Table 3. Heritability estimates for leaf macronutrient, Na concentrations and K/Na ratio ranged from 0.33 (Na and S) to 0.41 (K/Na ratio). If 10% of the RILs were selected from the population for high or low nutrient concentration, it resulted in a shift of the population mean in the respective direction to varying extents for each nutrient. When expressed as a percentage of the mean of an unselected population, the resultant shift was the smallest for P (6.3%), intermediate for Ca, Mg and S (>11%), high for K and Na (>15%), and highest for K/Na ratio (56%). A similar mean shift was observed when only 5% of the population was retained; however, a large increase occurred for K and K/Na ratio, because of a positive skewness in their distribution (Fig 1). For micronutrients, heritability estimates ranged from 0.27 (Mn and Cu) to 0.43 (B), and the percentage of predicted response was small for Fe and Cu, intermediate for Zn and Mn, and high for B.

**Table 3:** Estimates of narrow sense heritability (h^2^) and predicted selection response for five macro-, five micro-nutrient and Na concentrations and K/Na ratio on leaves of the RIL population under two selection intensities based on a combined data analysis over two seasons.

Nutrient	h^2^	Predicted selection response for selection intensity of			
		5%		10%	
		mg/kg	in % of the RIL mean	mg/kg	in % of the RIL mean
P	0.36 ± 0.04	265.3	7.5	222.3	6.3
K	0.39 ± 0.02	3519.8	22.2	2488.9	15.7
Ca	0.36 ± 0.03	2992.7	13.9	2398.2	11.1
Mg	0.37 ± 0.03	658.1	13.0	591.6	11.7
S	0.33 ± 0.03	1044.5	15.3	874.5	12.8
Na	0.33 ± 0.03	-179.9	-19.4	-163.2	-17.6
K/Na	0.41 ± 0.02	27.4	79.8	19.2	55.9
Fe	0.29 ± 0.05	4.17	5.1	3.39	4.2
Mn	0.27 ± 0.04	7.11	10.1	5.71	8.1
B	0.43 ± 0.01	14.93	22.6	12.49	18.9
Cu	0.27 ± 0.05	0.44	6.8	0.34	5.3
Zn	0.36 ± 0.03	1.56	9.4	1.28	7.7

### Interrelations of leaf nutrient concentrations

Pearson correlation coefficients for leaf macro-, micro-nutrient and Na concentrations and K/Na ratio are given in Table 4. Significant simple correlations existed in 44 of 66 pairs of nutrient combinations with the majority of them being positive. Interrelationships were more common between macronutrients, Na and K/Na ratio than between them and micronutrients (Table 4). For the interrelation of macronutrients, Na and K/Na ratio, nine pairs were positive with the strongest being among Ca, Mg and S, and between K and K/Na ratio (0.60 to 0.76, P<0.001), intermediate for Na with Ca, Mg and S, respectively (0.38 to 0.48, P<0.01), and the weakest for P with K and K/Na ratio, respectively (0.24 to 0.31, P<0.05). Seven pairs showed an inverse relation with the strongest between Na and K/Na (-0.69, P<0.001), intermediate for K/Na ratio with Ca and Mg, respectively, and for K with Na (-0.34 to -0.42, P<0.01), and weakest between K and Ca and between S and K/Na ratio (-0.24 to -0.26, P<0.05). The relation for K/Na ratio with macronutrients was consistent with that for K but opposite to that of Na with macronutrients, as expected from its mathematical derivation. Correlation was more common between Mn, B and Cu with the macronutrients, Na and K/Na ratio except K (Table 4). Most of those correlations were positive and weak but moderate between P and Zn, Ca, Mg and S with Mn (0.59 to 0.76, P<0.001). There were inverse correlations for B with P and K/Na ratio and also of Mn with K/Na ratio. In addition, Zn was positively associated with P, K and K/Na ratio. The opposite relationship was observed between K/Na ratio and Na with micronutrients. Fe was positively but weakly associated with Ca and S. Among micronutrients, only four combinations showed a positive relationship with a coefficient range from 0.38 (P<0.01) to 0.53 (P<0.001).

**Table 4:** Pearson correlation coefficients among macro-, micro-nutrient and Na concentrations and K/Na ratio on leaves of the RIL population based on a combined analysis of two seasons.

	K	Ca	Mg	S	Na	K/Na	Fe	Mn	B	Cu	Zn
P	0.24*	-0.10	-0.07	-0.01	-0.47***	0.31**	-0.08	0.03	-0.23*	0.30**	0.70***
K		-0.24*	-0.16	-0.04	-0.34**	0.60***	0.07	0.06	-0.15	-0.04	0.26*
Ca			0.76***	0.73***	0.48***	-0.42***	0.31**	0.76***	0.47***	0.38***	0.13
Mg				0.65***	0.47***	-0.42***	0.19	0.61***	0.40***	0.36**	0.08
S					0.38***	-0.26*	0.27*	0.59***	0.23*	0.42***	0.11
Na						-0.69***	0.13	0.33**	0.23*	0.10	-0.26*
K/Na							-0.05	-0.22*	-0.24*	-0.08	0.24*
Fe								0.42***	0.08	0.22	0.11
Mn									0.44***	0.38***	0.17
B										0.20	0.04
Cu											0.53***

*,**,*** indicate significance at P≤0.05, 0.01, 0.001, respectively.

n = 77.

### QTLs for leaf nutrient concentrations

Detailed QTL results are shown in Table 5 and S1 Fig. All variables except K detected at least one significant (LOD superior to permutation-based threshold) QTL. Twenty four significant QTLs were detected over the 12 variables, 10 QTLs for the macronutrients, Na and K/Na ratio (1 for P, 3 for Mg, 2 for Ca, 2 for Na, 1 for S and 1 for K/Na ratio) and accounted for about 12% to 17% trait variances; and 14 QTLs for the 5 micronutrients (2 for Fe, 3 for Mn, 3 for B, 2 for Cu and 4 for Zn) and accounted for 12% to 28% of trait variances. Additive effects were equally shared between the two parental effects except Ca, for which the *Gh* parent had positive effects. Twenty six additional putative QTLs (LOD>2.5) are also listed in Table 5 accounting for 8% to 11% of trait variance. The QTLs with highest LODs (≥7.0) were detected for Zn on chromosome 11 i.e. c11, Mn on c20 and Zn on c18. Chromosome regions with co-localised QTLs were on c14 between 76 and 88 cM (four significant QTLs for B, Zn, Na and K/Na, supplemented by three putative QTLs for each of Ca, K and Fe, for which effects were derived from GUA, except for K and K/Na ratio), c18 between 55 and 60 cM (three significant QTLs for Ca, Mg and Cu, for which effects were derived from GUA) and c22 between 43 and 49 cM (two significant QTLs for Fe and S, for which effects were derived from VH8). These co-localisations do not necessarily reflect the nature of correlations between traits. For example the two most correlated variables, Ca and Mn (r = 0.76, *P*<0.001), had two and three significant QTL, respectively, that did not co-localise. On the other hand, the correlations of Ca and Mg, S and Fe and Na and K/Na ratio corresponded with QTL co-localisations on c18, c22, and c14, respectively (Table 5, S1 Fig).

**Table 5:** Description of QTLs affecting leaf macro-, micro-nutrient and Na concentrations and K/Na ratio in the RIL population of Guazuncho 2 × VH8-4602 as detected by composite interval mapping using QTLCartographer based on the estimates of a two year combined analysis. “Signif” column indicates significant QTLs (LOD superior to permutation-based threshold). Putative QTLs with LOD>2.5 are also indicated. Positive sign of additive effect indicate that parent GUA increases the trait. LOD2L-LOD2R and LOD1L-LOD1R are the positions in cM of the drop-off in 2 or 1 LOD units from the LOD peak, respectively. R² is the proportion of the trait variance explained by the QTL.

Trait^a^	QTL name	Position		LOD	R²	Additivity	Signif	LOD2L	LOD1L	LOD1R	LOD2R
		Chrom	cM								
P	q[P]_2	2	39.1	3.3	0.10	-122.5		35.4	38.3	40.1	48.6
P	q[P]_5	5	95.9	4.62	0.17	-169	Signif	91.9	92.5	97.3	97.7
P	q[P]_14	14	30.9	2.8	0.09	100.8		24.3	27.6	33.4	35.9
P	q[P]_15	15	70.2	2.83	0.10	-131.8		55.9	68.2	70.2	70.2
P	q[P]_21	21	36.9	2.96	0.12	-115.9		32.9	32.9	42.9	46.4
K	q[K]_2	2	35.7	2.53	0.08	-1.054		33.9	34.1	36.8	38.0
K	q[K]_11	11	46.3	3.09	0.10	1.062		44.3	44.3	49.9	53.2
K	q[K]_14	14	81.4	3.69	0.12	-1.076		74.0	77.3	84.4	86.4
K	q[K]_22	22	31.3	2.69	0.09	-1.057		23.0	27.5	33.7	38.4
Ca	q[Ca]_14	14	78.8	3.33	0.11	1565		73.3	76.2	81.4	81.4
Ca	q[Ca]_18	18	54.7	3.8	0.13	1422	Signif	49.2	51.5	57.9	58.6
Ca	q[Ca]_24	24	29.8	3.68	0.13	1398	Signif	25.4	26.9	34.1	36.8
Mg	q[Mg]_1	1	34.3	4.43	0.20	-372	Signif	32.3	32.3	39.6	42.2
Mg	q[Mg]_11	11	6.8	2.92	0.09	278		2.1	4.2	13.3	16.7
Mg	q[Mg]_18	18	54.7	3.72	0.12	316	Signif	49.0	51.3	56.0	57.7
Mg	q[Mg]_22	22	0.0	3.91	0.13	307	Signif	0.0	0.0	4.2	4.2
S	q[S]_13	13	28.8	2.94	0.11	468		27.7	28.3	31.1	32.5
S	q[S]_22	22	43.4	3.7	0.14	-559	Signif	40.2	41.4	47.9	52.5
Na	q[Na]_1	1	3.8	3.74	0.14	137	Signif	0.1	1.5	6.6	9.3
Na	q[Na]_14	14	80.8	4.42	0.17	171	Signif	73.0	74.3	85.4	86.8
Na	q[Na]_18	18	10.6	3.06	0.13	-122		6.1	6.8	13.2	14.4
Na	q[Na]_24	24	45.8	3.02	0.11	121		41.4	43.5	48.3	50.4
K/Na	q[K/Na]_1	1	3.8	3.59	0.10	1.169		0.0	0.3	4.6	8.9
K/Na	q[K/Na]_3	3	9.0	3.07	0.09	-1.236		0.0	0.0	12.8	14.6
K/Na	q[K/Na]_5	5	98.9	2.82	0.08	-1.175		97.7	97.7	101.8	106.0
K/Na	q[K/Na]_11	11	65.3	3.03	0.08	-1.172		65	65.0	65.3	65.3
K/Na	q[K/Na]_14	14	78.8	4.79	0.14	-1.253	Signif	75.9	77.1	83.4	85.0
Fe	q[Fe]_8	8	48.4	4.91	0.17	3.89	Signif	47.1	47.3	50.2	53.8
Fe	q[Fe]_14	14	88.0	2.95	0.10	2.82		83.0	84.6	90.3	95.4
Fe	q[Fe]_22	22	49.3	5.24	0.18	-3.72	Signif	46.2	47.4	53.2	57.1
Mn	q[Mn]_18	18	15.5	3.11	0.10	3.71		12.2	13.7	18.5	20.6
Mn	q[Mn]_20	20	36.7	7.71	0.28	6.64	Signif	31.0	32.2	39.3	41.2
Mn	q[Mn]_21	21	121.7	3.5	0.18	4.86	Signif	110.5	113.5	128.9	129.9
Mn	q[Mn]_23	23	32	3.73	0.12	-4.06	Signif	28.4	29.5	34.6	35.6
Zn	q[Zn]_11	11	34.4	6.94	0.23	-1.16	Signif	34.0	34.1	37.0	38.0
Zn	q[Zn]_14	14	88.0	3.54	0.10	-0.76	Signif	81.4	83.7	91.0	94.3
Zn	q[Zn]_18	18	19.5	7.33	0.26	1.09	Signif	15.3	16.6	21.8	23.0
Zn	q[Zn]_26	26	54.6	3.97	0.13	-0.9	Signif	54.0	54.1	57.6	58.9
B	q[B]_10A	10A	22	5.52	0.18	-8.11	Signif	17.8	19.5	24.9	25.4
B	q[B]_10B	10B	36.9	2.78	0.09	5.59		36.2	36.2	43.0	46.2
B	q[B]_14	14	76.1	4.26	0.13	6.23	Signif	73.0	74.2	81.2	81.4
B	q[B]_19	19	44.9	3.94	0.12	-5.82	Signif	44.1	44.8	45.4	46.2
B	q[B]_25	25	10.3	3.23	0.11	5.43		0.3	4.5	14.3	16.3
Cu	q[Cu]_5	5	55.1	2.79	0.10	-0.25		50.9	52.2	57.3	57.3
Cu	q[Cu]_8	8	63.9	4.5	0.19	0.46	Signif	55.9	61.9	65.5	65.5
Cu	q[Cu]_12	12	58.4	2.6	0.11	0.25		50.0	54.3	60.7	62.3
Cu	q[Cu]_18	18	60.4	4.65	0.17	0.37	Signif	59.6	59.7	60.9	61.5

^a^ variables K and K/Na ratio were log-transformed before QTL analysis.

## Discussion

The conventional methods for estimating plant nutrient uptake and nutrient use efficiency require analysing nutrient concentrations in plant tissue samples or from whole plant samples [52]. However, it is difficult to screen breeding population particularly in early segregating generations, as the resources to do that are considerable and often impractical. Despite few studies on the relationship of leaf nutrient concentration and total uptake of nutrients in cotton, Na concentration in different plant parts was often measured and used as a surrogate for screening breeding germplasm or populations for salinity tolerance [24, 30, 34], a practise that is also common in the other crops [25, 27, 62]. When cotton was grown under irrigation with different K rates, total plant K uptake and K use efficiency were found to be significantly associated with K concentrations of plant stems, leaves, petioles and fruits; and the relationship with the concentration of top mature leaves was one of the strongest [11]. Total uptake for both macro- and micro-nutrients in cotton cultivars bred and released in the past in Australia was positively correlated with their concentrations in leaf samples collected during the same period as this study [37]. In our study, cotton leaves were one of the most discriminating tissues for the differences of parental nutrient status (Table 1); more interestingly, the variation within the RIL population for leaf nutrient concentrations was largely determined by genotype and with moderate heritability (Tables 2–3). Therefore, leaf nutrient concentration at early to peak flowering can be used as a simple and effective indicator for screening breeding populations. Others have also reported moderate heritability for shoot concentrations of K, Ca and K/Na ratio under saline conditions in cotton seedling stage [24, 34].

A pre-requisite for individual nutrients to support normal plant growth and development is that their concentrations remain above their critical value. The adequate range for most nutrients in cotton covers a wide range from at least two to ten-fold [8]. The Australian cotton production system relies on high inputs of N and P fertilisers and water to deliver high yields. Despite the RIL population being tested under such a high yielding management system, leaf concentrations for Ca and Mg were relatively high compared with published normal ranges for cotton [17], but generally lower than the ranges for the Australian controls that are adapted to that system (Fig 1). Leaf concentrations for B in the RILs were also generally higher than the published normal range [17] and generally also higher than the two local controls (Fig 2), with some RILs being high for B (up to 120 mg/kg), although no B toxicity symptoms were observed during the experiment. On average, concentrations of P, K, S, Zn and Cu were relative low but appeared to be adequate when compared with the concentrations of two local controls (Figs 1 and 2). Based on the nominated critical concentration for Cu in cotton leaves [63], this RIL population is clearly more than adequate. During the period of boll filling and maturation, the plant canopy of some RILs turned purple to red and even defoliated, a cotton premature senscense syndrome described previously in Australia [15]. Among these lines, some evidently had high Na concentration but low K/Na ratio—measures identified to be associated with the syndrome when grown in sodic soils in Australia [10, 15].

Segregation and predicted selection responses in this study suggested that favourable response to selection would be most likely for K, Na, K/Na ratio and B in this inter-specific RIL population (Table 3). For K, Na, and K/Na ratio, this was due to the large inherent parental difference, a large variability within the RIL population and the relatively high heritability of the traits in question (Fig 1, Table 3). However, a bias towards the low value *Gh* parent (GUA) in the population distribution for K concentration and K/Na ratio was observed, as was also seen for fibre properties and yield components in this same population [39–40]. This suggests that capturing favourable transgressive segregants of those traits would be more difficult than if they were normally distributed, so a relatively large population size would be needed in breeding to identify desired lines. For Na, on the other hand, the population distribution bias towards the low *Gb* parent (VH8) means that there should be a higher than expected frequency of lines with a desirable low leaf Na concentration in the population. Therefore, capturing segregants with low leaf Na concentrations should be straightforward, although the most useful RILs would be those with levels comparable to the *Gb* parent.

Unlike K, Na and K/Na ratio, the leaf B concentration had no clear parental difference, but there was still a large segregation in leaf B concentration across the RILs, possibly due to the allelic recombination during the construction and selection of the RIL population (Fig 2, Table 5). With relatively high heritability, selection response for B was high in this population (Table 3). B deficiency and toxicity in cotton do occur in light textured soils prone to leaching [64], and/or high yield systems where B removal is often higher after continuous cropping [3, 17]. B deficiency results in abnormal growth of fruit structures and increased fruit shedding with decreased cotton yields [64]. Therefore, B is an example where inter-specific crosses can offer nutritional traits to develop cotton better adapted to specific soil and management conditions.

The co-localisation of some QTLs for different macro- and micro-nutrients observed in this study (Table 5 and S1 Fig) was similar to that reported in *Medicago* for seed micronutrient concentrations [46]. Such phenomena mean that either the same alleles (i.e. pleiotropic effects) or multiple neighbouring alleles may govern the uptake, translocation and utilisation of some nutrients in cotton. It might also be indicative of differences in whole plant phenotypic traits contributing to leaf mineral concentration, such as differences between the parental genotypes and among the RILs, for their rooting characteristics, root nutrient uptake, and/or root-to-shoot transport. Although the root system of the two parents had not been studied, strong differences in the phenology and overall aerial morphological development of GUA and VH8 had been reported earlier [39]. The early and rapid root growth was reported important for high nutrient uptake after the onset of flowering in cotton [50–51] and large root system increased high K uptake in a K-use efficient cultivar under both irrigated and dryland conditions [14, 65]. Therefore, it is possible that screening for improved leaf nutrient status may indirectly change allelic combinations for root morphology and growth that favour nutrient uptake and water exploitation.

In the absence of a tetraploid cotton genome sequence it is difficult to identify the genes underlying any of the cotton nutrient QTLs. The recently published sequences of the diploid *Gossypium* species, *G*. *raimondii* [66] and *G*. *arboreum* [67], and the whole genome marker map [68] aligned to the *G*. *raimondii* reference genome, however, show that there is relatively good concordance in marker order between the ancestral diploid genomes and tetraploid cotton. According to some inference genes that may be present in any genetic interval of tetraploid cotton based on the genes present in *G*. *raimondii*, the cluster of macronutrient QTLs on c14 (equivalent to *G*. *raimondii* c5 [66]), for example, spans a large region that contains over 300 genes but this includes a couple of potential cation transporters or antiporters, and just flanking this region a cluster of three p-type ATPases (Gorai.005G220100.1–220300.1) (S2 Table)-that may be essential for the generation of the proton motive force necessary to drive transport of many different ions. The importance of alleles of those particular genes in nutrient transport would need to be confirmed in further studies.

The phenotypic correlations observed amongst leaf nutrient concentrations in this study highlight the importance of balanced nutrient status for cotton growth and development. Breeding could utilise such interactions to improve nutrient status in cotton either independently or simultaneously. For those with positive relationships, indirect selection can be employed, particularly for Ca, Mg and S, among which the correlations were strongest (Table 4). The common location of some significant QTLs for these nutrients suggests that the same or linked alleles may control their inheritance (Table 5), further dissection of those QTLs would be important for defining the correct breeding strategy as well as for applying marker assisted selection. On the other hand, for those pairs with moderate relationships, such as between macro- and micro-nutrients or among micronutrients (Table 4), directional selection for macro- or micro-nutrients may lead to some unexpected response of related micronutrients in this population. When such responses force cotton plants to uptake and accumulate too little or too much related micronutrients, it may result in plant nutrient deficiency or toxicity. Therefore, some caution should be taken when applying indirect selection for improving cotton nutrient balance and status.

The inheritance of leaf Na concentration and its relation with the macronutrients in this study are of particular interest in terms of the usefulness of these inter-specific RILs for enhancing sodicity tolerance in Australian *Gh* cultivars. The selection for low Na concentration and high K/Na ratio would increase uptake and utilisation of both P and K (as they are negatively correlated with Na)(Table 4), which agrees with local studies that the high Na uptake of cotton in sodic soils reduced both P and K uptake [10, 15, 69]. QTL effects for Na and K/Na ratio also suggest the introgression of relatively few *Gb* alleles into a *Gh* background would lower Na concentrations and maintain high K/Na ratios at least in cotton leaves (Tables 1 and 5). *Gb* and the better inter-specific derived RIL lines all possessed inherent low sodium uptake and accumulation (Table 1, Fig 1) (consistent with the previous studies [33, 34]), so choosing better sodium tolerant *Gh* sources as breeding parents becomes crucial for developing new *Gh* cultivars with better tolerance to soil sodicity when interspecific breeding is employed. The local control *Gh* cultivar, Sicot 75 used in this study already exhibited much lower accumulated Na in leaves than the *Gh* RIL parent, GUA (Fig 1). This is not surprising as that cultivar was bred for local often sodic conditions, and has shown itself to be well adapted and have sufficient tolerance to sodic soil like many other locally bred cultivars [69]. It should be a suitable *Gh* parent for in crossing with some of the better RILs. Furthermore, high leaf K/Na ratios could be used as the selection criterion to screen large numbers of individuals in early generation breeding material [70] following similar practices used in other field crops [62]. It is also a more cost effective approach compared to yield phenotyping under saline conditions. In sodic Australian soils, cotton uptake for P has been reported to be more compromised than for K [10]. In this RIL population, there was, however, a strong relationship between P/Na and K/Na ratios (r = 0.86, P<0.001) when P/Na ratio was analyed, therefore, the aforementioned efforts should indirectly enhance cotton P uptake and utilisation and hence its overall adaptability to sodic soil conditions.

Although not the only mechansim for salt tolerance in plants, there is convincing evidence to suggest that selecting for exclusion of Na from the above ground tissues is a valid approach for enhancing salinity/sodicity tolerance in cotton and many crops [70]. Some *Gb* cultivars have been reported to be more effective in limiting Na accumulation in leaves and to be more salt and sodic tolerant than high Na accumulators [30, 34] and this is also true for the VH8 parent of this RIL population. Further more, VH8 accumulated much lower levels of Na in the leaf, petiole and stem than does GUA, but the roots accumulate just as high or even higher levels of Na (Table 1), suggesting that it is not solely Na exclusion from the root *per se*, the mechanism similar to that reported in some salt tolerant lines of Durum wheat, for example, where Na^+^ transporters *Nax1* and *Nax2* actively transport Na^+^ back out of the xylem to prevent subsequent translocation to other critical parts of the plant [71], is likely to be involved, but this will need to be explored in more detail in cotton.

P and K are two important nutrients along with nitrogen and their positive relation is understandable, as cotton’s requirement for those nutrients increases with increased growth and yield. The inverse relation of K or K/Na with Ca and Mg confirmed the suppressive effect of Ca and Mg over K uptake in cotton and other field crops [6, 13]. Cotton soils in Australia are generally rich in Ca, Mg and S [3], positive relations between these nutrients (Table 4) and many yield components (S3 Table) confirm the importance of maintaining their nutritional levels in soils for high productivity. The relationships of Na with K and Ca in this study were consistent with those reported previously [72] concerning cotton root uptake for Na, K and Ca at the seedling stage. The positive and strong relationship between P and Zn suggested a synergistic interaction of these two nutrients, which is opposite to the previous result in old cotton leaves where Zn deficiency increased P accumulation to toxic levels [73]. Among the micronutrients, the positive relation between Mn and B was in agreement with previous studies [74] but B and Zn were not correlated in this population.

This study demonstrates that genetic diversity of two cultivated cotton tetraploid species can be exploited to develop germplasm with improved cotton nutrient status. Allelic introgression and/or recombination between two species are likely reasons behind observed heritable variation of leaf nutrient concentrations within the RIL population. The interrelations of nutrient concentrations with yield components or fibre properties (S3 and S4 Tables) do not suggest major genetic obstacles for combining improved plant nutrient status with high yielding or desirable fibre properties. Given cotton cultivars with better nutrient uptake and use efficiency and/or tolerance to soil nutrient stresses would improve overall fertiliser use efficiency in farms and also reduce soil-related nutrient problems, this study provides evidence on how inter-specific breeding can offer novel genetic variations useful to develop highly performing and adapted cotton.

## Supporting Information

S1 Fig Genetic map of Guazuncho 2 × VH8-4602 showing the locations of QTLs affecting macro-, micro-nutrient and Na concentrations and K/Na ratio in leaves. QTLs shown as black and hashed boxes represent significant (LOD superior to permutation-based threshold) and putative QTLs (LOD>2.5), respectively. Map position likelihood confidence intervals are shown as boxes (1LOD drop-off) and bars (2LOD drop-off).
(DOCX)

S1 Table Correlation coefficients between the two years of leaf nutrient concentration estimates of the RIL population (see the values in bold along the diagonal of the table). (DOCX)

S2 Table Genes in *G*. *raimondii* that are located in the interval spanning the cluster of nutrient concentration QTLs mapped in the *G*. *hirsutum* × *G*. *barbadense* RIL population. *G*. *raimondii* Chromosome 5 is highly syntenic to the *G*. *hirsutum* Chromosome 14 belonging to the Dt sub-genome. Genes potentially involved in ion transport are indicated in red, while the region spanning the most central three markers in the interval are marked in yellow.
(DOCX)

S3 Table Pearson correlation coefficients of five macronutrient, Na concentrations and K/Na ratio in leaves with yield components and fibre properties in the RIL population based on a combined data analysis over two seasons. (DOCX)

S4 Table Pearson correlation coefficients of five micronutrient concentrations in leaves with yield components and fibre properties for the RIL population based on a combined data analysis over two seasons. (DOCX)
