# Supplementary material for: Inheritance and QTL Mapping of Leaf Nutrient Concentration in a Cotton Inter-Specific Derived RIL Population
Source: PLoS One. 2015 May 28;10(5):e0128100. doi: 10.1371/journal.pone.0128100 (PMC4447399; doi:10.1371/journal.pone.0128100)
Supplement: S1 Fig — QTLs shown as black and hashed boxes represent significant (LOD superior to permutation-based threshold) and putative QTLs (LOD>2.5), respectively. Map position likelihood confidence intervals are shown as boxes (1LOD drop-off) and bars (2LOD drop-off). (DOCX) [file pone.0128100.s001.docx]

**S1 Fig. Genetic map of Guazuncho 2 × VH8-4602 showing the locations of QTLs affecting macro-, micro-nutrient and Na concentrations and K/Na ratio in leaves.** QTLs shown as black and hashed boxes represent significant (LOD superior to permutation-based threshold) and putative QTLs (LOD>2.5), respectively. Map position likelihood confidence intervals are shown as boxes (1LOD drop-off) and bars (2LOD drop-off).
