# Supplementary material for: Inheritance and QTL Mapping of Leaf Nutrient Concentration in a Cotton Inter-Specific Derived RIL Population
Source: PLoS One. 2015 May 28;10(5):e0128100. doi: 10.1371/journal.pone.0128100 (PMC4447399; doi:10.1371/journal.pone.0128100)
Supplement: S1 Table — (DOCX) [file pone.0128100.s002.docx]

**S1 Table. Correlation coefficients between the two years of leaf nutrient concentration estimates of the RIL population** (see the values in bold along the diagonal of the table).

|  |  | 2008/2009 | | | | | | | | | | | | |
| --- | --- | --- | --- | --- | --- | --- | --- | --- | --- | --- | --- | --- | --- | --- |
|  |  | P | K | Ca | Mg | S | Na | K/Na | Fe | Mn | B | Cu | Zn |  |
| 2007/08 | P | **0.86** | 0.24 | -0.22 | -0.10 | -0.04 | -0.42 | 0.24 | -0.11 | -0.10 | -0.29 | 0.24 | 0.61 |  |
|  | K | 0.19 | **0.74** | -0.27 | -0.13 | -0.06 | -0.22 | 0.37 | 0.05 | -0.09 | -0.19 | 0.00 | 0.25 |  |
|  | Ca | -0.07 | -0.26 | **0.75** | 0.59 | 0.53 | 0.36 | -0.27 | 0.10 | 0.64 | 0.34 | 0.24 | 0.17 |  |
|  | Mg | -0.11 | -0.27 | 0.68 | **0.81** | 0.58 | 0.41 | -0.29 | 0.03 | 0.60 | 0.34 | 0.24 | 0.15 |  |
|  | S | -0.05 | -0.06 | 0.55 | 0.47 | **0.70** | 0.29 | -0.16 | 0.08 | 0.48 | 0.21 | 0.25 | 0.12 |  |
|  | Na | -0.37 | -0.38 | 0.50 | 0.48 | 0.40 | **0.83** | -0.56 | 0.10 | 0.40 | 0.25 | 0.09 | -0.06 |  |
|  | K/Na | 0.29 | 0.65 | -0.45 | -0.50 | -0.34 | -0.72 | **0.91** | 0.00 | -0.33 | -0.30 | -0.08 | 0.15 |  |
|  | Fe | -0.08 | -0.03 | 0.32 | 0.22 | 0.27 | 0.12 | -0.06 | **0.74** | 0.40 | 0.01 | 0.21 | 0.10 |  |
|  | Mn | 0.03 | -0.04 | 0.48 | 0.42 | 0.41 | 0.26 | -0.17 | 0.11 | **0.60** | 0.26 | 0.18 | 0.15 |  |
|  | B | -0.14 | -0.15 | 0.37 | 0.34 | 0.18 | 0.21 | -0.22 | 0.08 | 0.45 | **0.89** | 0.23 | 0.11 |  |
|  | Cu | 0.15 | -0.06 | 0.30 | 0.29 | 0.34 | 0.11 | -0.06 | 0.12 | 0.39 | 0.10 | **0.67** | 0.39 |  |
|  | Zn | 0.65 | 0.20 | 0.04 | 0.01 | 0.10 | -0.25 | 0.20 | 0.05 | 0.11 | -0.02 | 0.46 | **0.95** |  |
