# Supplementary material for: Inheritance and QTL Mapping of Leaf Nutrient Concentration in a Cotton Inter-Specific Derived RIL Population
Source: PLoS One. 2015 May 28;10(5):e0128100. doi: 10.1371/journal.pone.0128100 (PMC4447399; doi:10.1371/journal.pone.0128100)
Supplement: S2 Table — G. raimondii Chromosome 5 is highly syntenic to the G. hirsutum Chromosome 14 belonging to the Dt sub-genome. Genes potentially involved in ion transport are indicated in red, while the region spanning the most central three markers in the interval are marked in yellow. (DOCX) [file pone.0128100.s003.docx]

**S2 Table. Genes in *G. raimondii* that are located in the interval spanning the cluster of nutrient concentration QTLs mapped in the *G. hirsutum* × *G. barbadense* RIL population.** *G. raimondii* Chromosome 5 is highly syntenic to the *G. hirsutum* Chromosome 14 belonging to the D^t^ sub-genome. Genes potentially involved in ion transport are indicated in red, while the region spanning the most central three markers in the interval are marked in yellow.

| **Locus ID** | **Chr.** | **Start** | **End** | **Function** | **3 markers most central to the interval** |
| --- | --- | --- | --- | --- | --- |
| Gorai.005G212700.1 | Chr05 | 59501910 | 59503066 | GEM-like protein |  |
| Gorai.005G212800.1 | Chr05 | 59505346 | 59506566 | GEM-like protein |  |
| Gorai.005G212900.1 | Chr05 | 59511622 | 59516895 | Duplicated homeodomain-like superfamily protein |  |
| Gorai.005G213000.1 | Chr05 | 59538275 | 59542556 | Calcium-binding EF hand family protein |  |
| Gorai.005G213100.1 | Chr05 | 59542581 | 59547020 | 5-oxoprolinase |  |
| Gorai.005G213200.1 | Chr05 | 59580614 | 59583022 | unknown protein |  |
| Gorai.005G213300.1 | Chr05 | 59583223 | 59587669 | Mitochondrial import receptor subunit TOM40 homolog |  |
| Gorai.005G213400.1 | Chr05 | 59588117 | 59596596 | SNF2 domain-containing protein / helicase domain-containing protein / zinc finger protein-related |  |
| Gorai.005G213500.1 | Chr05 | 59601363 | 59601566 | unknown protein |  |
| Gorai.005G213600.1 | Chr05 | 59622688 | 59628565 | Endosomal targeting BRO1-like domain-containing protein |  |
| Gorai.005G213700.1 | Chr05 | 59627833 | 59631936 | Phytochrome-associated serine/threonine-protein phosphatase |  |
| Gorai.005G213800.1 | Chr05 | 59654147 | 59657600 | unknown protein |  |
| Gorai.005G213900.1 | Chr05 | 59657958 | 59664949 | Lipoyl synthase 1, chloroplastic |  |
| Gorai.005G214000.1 | Chr05 | 59669895 | 59673377 | Pyruvate kinase, cytosolic isozyme |  |
| Gorai.005G214100.1 | Chr05 | 59675936 | 59678530 | Alpha-galactosidase |  |
| Gorai.005G214200.1 | Chr05 | 59679447 | 59684136 | ABC transporter B family member |  |
| Gorai.005G214300.1 | Chr05 | 59686674 | 59688001 | ferredoxin/thioredoxin reductase subunit A (variable subunit) 2 |  |
| Gorai.005G214400.1 | Chr05 | 59694263 | 59702365 | Katanin p80 WD40 repeat-containing subunit B1 homolog |  |
| Gorai.005G214500.1 | Chr05 | 59705374 | 59708636 | Protein of unknown function (DUF789) |  |
| Gorai.005G214600.1 | Chr05 | 59707992 | 59710743 | Protein of unknown function (DUF3531) |  |
| Gorai.005G214700.1 | Chr05 | 59712451 | 59715034 | Aconitase/3-isopropylmalate dehydratase protein |  |
| Gorai.005G214800.1 | Chr05 | 59717292 | 59721547 | Ankyrin repeat domain-containing protein |  |
| Gorai.005G214900.1 | Chr05 | 59724891 | 59728002 | UDP-galactose transporter |  |
| Gorai.005G215000.1 | Chr05 | 59731665 | 59736156 | Histone deacetylase |  |
| Gorai.005G215100.1 | Chr05 | 59738916 | 59739668 | delay of germination 1 |  |
| Gorai.005G215200.1 | Chr05 | 59740819 | 59744656 | Clathrin adaptor complex small chain family protein |  |
| Gorai.005G215300.1 | Chr05 | 59768292 | 59768744 | RING/U-box superfamily protein |  |
| Gorai.005G215400.1 | Chr05 | 59776390 | 59778165 | Putative DNA-binding protein ESCAROLA |  |
| Gorai.005G215500.1 | Chr05 | 59813229 | 59815800 | 3-hydroxy-3-methylglutaryl-coenzyme A reductase |  |
| Gorai.005G215600.1 | Chr05 | 59823725 | 59825916 | 3-hydroxy-3-methylglutaryl-coenzyme A reductase |  |
| Gorai.005G215700.1 | Chr05 | 59831119 | 59833745 | 3-hydroxy-3-methylglutaryl-coenzyme A reductase |  |
| Gorai.005G215800.1 | Chr05 | 59855232 | 59857766 | 3-hydroxy-3-methylglutaryl-coenzyme A reductase |  |
| Gorai.005G215900.1 | Chr05 | 59878724 | 59887568 | SEC7-like guanine nucleotide exchange family protein |  |
| Gorai.005G216000.1 | Chr05 | 59901496 | 59903005 | Vacuolar sorting protein 9 (VPS9) domain |  |
| Gorai.005G216100.1 | Chr05 | 59903983 | 59905842 | unknown protein |  |
| Gorai.005G216200.1 | Chr05 | 59910706 | 59913679 | Aminoacylase-1 |  |
| Gorai.005G216300.1 | Chr05 | 59914276 | 59918305 | unknown protein |  |
| Gorai.005G216400.1 | Chr05 | 59928696 | 59929178 | unknown protein |  |
| Gorai.005G216500.1 | Chr05 | 59941505 | 59946021 | Calcium-dependent protein kinase |  |
| Gorai.005G216600.1 | Chr05 | 59956340 | 59960838 | Monosaccharide-sensing protein |  |
| Gorai.005G216700.1 | Chr05 | 59961247 | 59962576 | Homoserine kinase |  |
| Gorai.005G216800.1 | Chr05 | 59963191 | 59966338 | Probable anion transporter |  |
| Gorai.005G216900.1 | Chr05 | 60000517 | 60001139 | XH/XS domain-containing protein |  |
| Gorai.005G217000.1 | Chr05 | 60001764 | 60004284 | ARM repeat superfamily protein |  |
| Gorai.005G217100.1 | Chr05 | 60006216 | 60008922 | Transmembrane amino acid transporter family protein |  |
| Gorai.005G217200.1 | Chr05 | 60013235 | 60014931 | EXORDIUM like 5 |  |
| Gorai.005G217300.1 | Chr05 | 60035993 | 60039318 | Protein kinase superfamily protein |  |
| Gorai.005G217400.1 | Chr05 | 60047648 | 60049783 | xylem NAC domain 1 |  |
| Gorai.005G217500.1 | Chr05 | 60056591 | 60057384 | ATP synthase epsilon chain, chloroplastic |  |
| Gorai.005G217600.1 | Chr05 | 60061316 | 60063623 | L-type lectin-domain containing receptor kinase IV.2 |  |
| Gorai.005G217700.1 | Chr05 | 60063908 | 60070310 | Dynamin-like protein ARC5 |  |
| Gorai.005G217800.1 | Chr05 | 60075603 | 60078158 | Zinc finger C-x8-C-x5-C-x3-H type family protein |  |
| Gorai.005G217900.1 | Chr05 | 60078275 | 60081525 | loricrin-related |  |
| Gorai.005G218000.1 | Chr05 | 60082707 | 60086573 | Beta-xylosidase/alpha-L-arabinofuranosidase |  |
| Gorai.005G218100.1 | Chr05 | 60131834 | 60133076 | unknown protein |  |
| Gorai.005G218200.1 | Chr05 | 60135781 | 60143685 | BRASSINOSTEROID INSENSITIVE 1-associated receptor kinase |  |
| Gorai.005G218300.1 | Chr05 | 60164399 | 60169598 | Aminotransferase ALD1 |  |
| Gorai.005G218400.1 | Chr05 | 60169674 | 60172834 | F-box/kelch-repeat protein At1g22040 |  |
| Gorai.005G218500.1 | Chr05 | 60189589 | 60191406 | Heavy metal transport/detoxification superfamily protein |  |
| Gorai.005G218600.1 | Chr05 | 60192606 | 60196335 | High affinity sulfate transporter |  |
| Gorai.005G218700.1 | Chr05 | 60196057 | 60197970 | Bifunctional inhibitor/lipid-transfer protein/seed storage 2S albumin superfamily protein |  |
| Gorai.005G218800.1 | Chr05 | 60203158 | 60211090 | Protein phosphatase 2C family protein |  |
| Gorai.005G218900.1 | Chr05 | 60219099 | 60220584 | basic helix-loop-helix (bHLH) DNA-binding superfamily protein |  |
| Gorai.005G219000.1 | Chr05 | 60227012 | 60228254 | Chlorophyll a-b binding protein CP24 10A, chloroplastic |  |
| Gorai.005G219100.1 | Chr05 | 60230885 | 60232318 | Peroxidase N1 |  |
| Gorai.005G219200.1 | Chr05 | 60239118 | 60240559 | Cationic peroxidase |  |
| Gorai.005G219300.1 | Chr05 | 60255649 | 60258073 | ATP synthase subunit d, mitochondrial |  |
| Gorai.005G219400.1 | Chr05 | 60258497 | 60260664 | Cationic peroxidase |  |
| Gorai.005G219500.1 | Chr05 | 60264128 | 60266237 | Probable inactive receptor kinase RLK902 |  |
| Gorai.005G219600.1 | Chr05 | 60283567 | 60284934 | annexin 2 |  |
| Gorai.005G219700.1 | Chr05 | 60285001 | 60288238 | cAMP-regulated phosphoprotein 19-related protein |  |
| Gorai.005G219800.1 | Chr05 | 60292566 | 60298522 | CD2-binding protein-related |  |
| Gorai.005G219900.1 | Chr05 | 60299090 | 60301254 | unknown protein |  |
| Gorai.005G220000.1 | Chr05 | 60300057 | 60303921 | ortholog of human splicing factor SC35 |  |
| Gorai.005G220100.1 | Chr05 | 60313710 | 60314544 | P-type ATP-ase 1 |  |
| Gorai.005G220200.1 | Chr05 | 60316083 | 60316582 | P-type ATP-ase 1 |  |
| Gorai.005G220300.1 | Chr05 | 60317812 | 60325682 | P-type ATP-ase 1 |  |
| Gorai.005G220400.1 | Chr05 | 60326314 | 60329009 | Alternative oxidase, mitochondrial |  |
| Gorai.005G220500.1 | Chr05 | 60330995 | 60333324 | Alternative oxidase, mitochondrial |  |
| Gorai.005G220600.1 | Chr05 | 60341564 | 60349789 | Calmodulin-binding transcription activator |  |
| Gorai.005G220700.1 | Chr05 | 60350693 | 60353743 | tobamovirus multiplication 1 |  |
| Gorai.005G220800.1 | Chr05 | 60358432 | 60359220 | Polynucleotidyl transferase, ribonuclease H-like superfamily protein |  |
| Gorai.005G220900.1 | Chr05 | 60362340 | 60364699 | Metacaspase-1 |  |
| Gorai.005G221000.1 | Chr05 | 60363762 | 60367256 | unknown protein |  |
| Gorai.005G221100.1 | Chr05 | 60370765 | 60375843 | Outer envelope protein 64, mitochondrial |  |
| Gorai.005G221200.1 | Chr05 | 60378264 | 60382107 | Probable protein phosphatase 2C |  |
| Gorai.005G221300.1 | Chr05 | 60388697 | 60393356 | AGAMOUS-like 20 |  |
| Gorai.005G221400.1 | Chr05 | 60394204 | 60396283 | metacaspase 1 |  |
| Gorai.005G221500.1 | Chr05 | 60403198 | 60407125 | methyl-CPG-binding domain 8 |  |
| Gorai.005G221600.1 | Chr05 | 60424839 | 60427434 | ABC transporter G family member | NAU3598 (HDC_c14_37.4) |
| Gorai.005G221700.1 | Chr05 | 60431735 | 60433036 | white-brown complex homolog protein 11 |  |
| Gorai.005G221800.1 | Chr05 | 60437644 | 60438929 | white-brown complex homolog protein 11 |  |
| Gorai.005G221900.1 | Chr05 | 60453996 | 60456300 | ABC transporter G family member |  |
| Gorai.005G222000.1 | Chr05 | 60488619 | 60489633 | Chloroplast Ycf2;ATPase, AAA type, core |  |
| Gorai.005G222100.1 | Chr05 | 60489413 | 60492272 | ABC transporter G family member |  |
| Gorai.005G222200.1 | Chr05 | 60506538 | 60507075 | Uncharacterised protein family (UPF0497) |  |
| Gorai.005G222300.1 | Chr05 | 60512471 | 60512787 | Bifunctional inhibitor/lipid-transfer protein/seed storage 2S albumin superfamily protein |  |
| Gorai.005G222400.1 | Chr05 | 60520371 | 60523437 | white-brown complex homolog protein 11 |  |
| Gorai.005G222500.1 | Chr05 | 60526171 | 60529359 | UbiA prenyltransferase family protein |  |
| Gorai.005G222600.1 | Chr05 | 60531543 | 60533862 | 14-3-3 protein |  |
| Gorai.005G222700.1 | Chr05 | 60533349 | 60535535 | unknown protein |  |
| Gorai.005G222800.1 | Chr05 | 60537733 | 60541831 | LMBR1-like membrane protein |  |
| Gorai.005G222900.1 | Chr05 | 60542535 | 60545850 | phytosulfokin receptor 1 |  |
| Gorai.005G223000.1 | Chr05 | 60547401 | 60548054 | unknown protein |  |
| Gorai.005G223100.1 | Chr05 | 60549099 | 60549857 | phytosulfokin receptor 1 |  |
| Gorai.005G223200.1 | Chr05 | 60549954 | 60550888 | unknown protein |  |
| Gorai.005G223300.1 | Chr05 | 60550967 | 60555266 | hAT dimerisation domain-containing protein |  |
| Gorai.005G223400.1 | Chr05 | 60554699 | 60556446 | unknown protein |  |
| Gorai.005G223500.1 | Chr05 | 60564320 | 60567844 | 2-oxoglutarate/malate translocator, chloroplastic |  |
| Gorai.005G223600.1 | Chr05 | 60571977 | 60574020 | unknown protein |  |
| Gorai.005G223700.1 | Chr05 | 60579830 | 60584397 | Riboflavin biosynthesis protein ribBA, chloroplastic |  |
| Gorai.005G223800.1 | Chr05 | 60584928 | 60585900 | arabinogalactan protein 1 |  |
| Gorai.005G223900.1 | Chr05 | 60588300 | 60589046 | unknown protein |  |
| Gorai.005G224000.1 | Chr05 | 60593988 | 60597807 | unknown protein |  |
| Gorai.005G224100.1 | Chr05 | 60606937 | 60608229 | Vinorine synthase |  |
| Gorai.005G224200.1 | Chr05 | 60621712 | 60624865 | Root phototropism protein |  |
| Gorai.005G224300.1 | Chr05 | 60629589 | 60630653 | sequence-specific DNA binding transcription factors;transcription regulators |  |
| Gorai.005G224400.1 | Chr05 | 60630955 | 60631243 | conserved peptide upstream open reading frame 37 |  |
| Gorai.005G224500.1 | Chr05 | 60638020 | 60639316 | Probable mitochondrial 2-oxoglutarate/malate carrier protein |  |
| Gorai.005G224600.1 | Chr05 | 60641070 | 60643348 | Peptidyl-prolyl cis-trans isomerase FKBP12 |  |
| Gorai.005G224700.1 | Chr05 | 60652998 | 60654520 | unknown protein |  |
| Gorai.005G224800.1 | Chr05 | 60677999 | 60678822 | unknown protein |  |
| Gorai.005G224900.1 | Chr05 | 60691359 | 60694133 | unknown protein |  |
| Gorai.005G225000.1 | Chr05 | 60696717 | 60697948 | hydroxyproline-rich glycoprotein family protein |  |
| Gorai.005G225100.1 | Chr05 | 60719669 | 60721182 | unknown protein |  |
| Gorai.005G225200.1 | Chr05 | 60738282 | 60739696 | unknown protein |  |
| Gorai.005G225300.1 | Chr05 | 60756855 | 60757949 | unknown protein |  |
| Gorai.005G225400.1 | Chr05 | 60759320 | 60762196 | beta carbonic anhydrase 5 |  |
| Gorai.005G225500.1 | Chr05 | 60804068 | 60816423 | MADS-box protein SVP |  |
| Gorai.005G225600.1 | Chr05 | 60817988 | 60822408 | Rab GDP dissociation inhibitor alpha |  |
| Gorai.005G225700.1 | Chr05 | 60823568 | 60832994 | Cysteine proteinases superfamily protein |  |
| Gorai.005G225800.1 | Chr05 | 60833621 | 60834847 | Ribosomal protein L10 family protein |  |
| Gorai.005G225900.1 | Chr05 | 60838632 | 60843958 | synaptotagmin A |  |
| Gorai.005G226000.1 | Chr05 | 60845191 | 60850327 | ZIM-like 1 |  |
| Gorai.005G226100.1 | Chr05 | 60850584 | 60859150 | Dynamin-related protein 3A |  |
| Gorai.005G226200.1 | Chr05 | 60861846 | 60862279 | regulatory particle triple-A ATPase 6A |  |
| Gorai.005G226300.1 | Chr05 | 60866030 | 60867957 | Cox19-like CHCH family protein |  |
| Gorai.005G226400.1 | Chr05 | 60867062 | 60871691 | Class I glutamine amidotransferase-like superfamily protein |  |
| Gorai.005G226500.1 | Chr05 | 60882600 | 60882893 | unknown protein |  |
| Gorai.005G226600.1 | Chr05 | 60884548 | 60885945 | unknown protein |  |
| Gorai.005G226700.1 | Chr05 | 60920410 | 60923330 | HVA22 homologue A |  |
| Gorai.005G226800.1 | Chr05 | 60923990 | 60930360 | Methylmalonate-semialdehyde dehydrogenase [acylating], mitochondrial |  |
| Gorai.005G226900.1 | Chr05 | 60931213 | 60940609 | aldehyde dehydrogenase 6B2 |  |
| Gorai.005G227000.1 | Chr05 | 60955390 | 60957774 | Probable pectate lyase P59 |  |
| Gorai.005G227100.1 | Chr05 | 61007147 | 61007758 | unknown protein |  |
| Gorai.005G227200.1 | Chr05 | 61016329 | 61025043 | AGAMOUS-like 44 |  |
| Gorai.005G227300.1 | Chr05 | 61039930 | 61048030 | Oligopeptide transporter |  |
| Gorai.005G227400.1 | Chr05 | 61061493 | 61065075 | Magnesium transporter CorA-like family protein |  |
| Gorai.005G227500.1 | Chr05 | 61067503 | 61069744 | DNA polymerase V family |  |
| Gorai.005G227600.1 | Chr05 | 61073284 | 61073558 | ribosomal protein S2 |  |
| Gorai.005G227700.1 | Chr05 | 61073819 | 61074696 | unknown protein |  |
| Gorai.005G227800.1 | Chr05 | 61083672 | 61085543 | Octicosapeptide/Phox/Bem1p family protein |  |
| Gorai.005G227900.1 | Chr05 | 61087501 | 61090378 | APO protein 3, mitochondrial |  |
| Gorai.005G228000.1 | Chr05 | 61090785 | 61093900 | ATP-dependent Clp protease proteolytic subunit 6, chloroplastic |  |
| Gorai.005G228100.1 | Chr05 | 61094128 | 61095372 | Mediator of RNA polymerase II transcription subunit |  |
| Gorai.005G228200.1 | Chr05 | 61096898 | 61097998 | unknown protein |  |
| Gorai.005G228300.1 | Chr05 | 61101904 | 61103037 | hydroxyproline-rich glycoprotein family protein |  |
| Gorai.005G228400.1 | Chr05 | 61107181 | 61128884 | Phosphatidylinositol 4-kinase alpha |  |
| Gorai.005G228500.1 | Chr05 | 61132359 | 61134631 | Polyol transporter |  |
| Gorai.005G228600.1 | Chr05 | 61164636 | 61168771 | HVA22-like protein k |  |
| Gorai.005G228700.1 | Chr05 | 61167092 | 61170578 | Urea-proton symporter DUR3 |  |
| Gorai.005G228800.1 | Chr05 | 61171335 | 61173681 | Quinone reductase family protein |  |
| Gorai.005G228900.1 | Chr05 | 61181694 | 61183370 | UPF0496 protein At4g34320 |  |
| Gorai.005G229000.1 | Chr05 | 61185583 | 61189024 | Cytochrome c biogenesis protein CCS1, chloroplastic |  |
| Gorai.005G229100.1 | Chr05 | 61188100 | 61192307 | 4-hydroxy-3-methylbut-2-enyl diphosphate reductase, chloroplastic |  |
| Gorai.005G229200.1 | Chr05 | 61196184 | 61202784 | Protein TIC 62, chloroplastic |  |
| Gorai.005G229300.1 | Chr05 | 61203756 | 61207244 | unknown protein |  |
| Gorai.005G229400.1 | Chr05 | 61206713 | 61211364 | Inositol 1,3,4-trisphosphate 5/6-kinase family protein |  |
| Gorai.005G229500.1 | Chr05 | 61223772 | 61225506 | unknown protein |  |
| Gorai.005G229600.1 | Chr05 | 61225507 | 61227836 | Tubulin alpha-4 chain |  |
| Gorai.005G229700.1 | Chr05 | 61229242 | 61234475 | unknown protein |  |
| Gorai.005G229800.1 | Chr05 | 61240022 | 61241359 | Salutaridinol 7-O-acetyltransferase |  |
| Gorai.005G229900.1 | Chr05 | 61249899 | 61250572 | phytosulfokine 6 precursor |  |
| Gorai.005G230000.1 | Chr05 | 61259677 | 61261955 | unknown protein |  |
| Gorai.005G230100.1 | Chr05 | 61275599 | 61276332 | Uncharacterised conserved protein UCP031279 |  |
| Gorai.005G230200.1 | Chr05 | 61278904 | 61280227 | unknown protein |  |
| Gorai.005G230300.1 | Chr05 | 61296746 | 61298969 | CYCLIN D1;1 |  |
| Gorai.005G230400.1 | Chr05 | 61314212 | 61314637 | Bifunctional inhibitor/lipid-transfer protein/seed storage 2S albumin superfamily protein |  |
| Gorai.005G230500.1 | Chr05 | 61316131 | 61316858 | Bifunctional inhibitor/lipid-transfer protein/seed storage 2S albumin superfamily protein |  |
| Gorai.005G230600.1 | Chr05 | 61320201 | 61323854 | Nucleobase-ascorbate transporter |  |
| Gorai.005G230700.1 | Chr05 | 61336635 | 61338345 | proline-rich family protein |  |
| Gorai.005G230800.1 | Chr05 | 61340479 | 61344536 | Telomere repeat-binding factor |  |
| Gorai.005G230900.1 | Chr05 | 61346739 | 61348620 | GATA type zinc finger transcription factor family protein |  |
| Gorai.005G231000.1 | Chr05 | 61352925 | 61356749 | ADP-ribosylation factor-like protein 8B |  |
| Gorai.005G231100.1 | Chr05 | 61359440 | 61362726 | Glycosyl hydrolase superfamily protein |  |
| Gorai.005G231200.1 | Chr05 | 61366973 | 61374301 | Glycosyl hydrolase family 10 protein |  |
| Gorai.005G231300.1 | Chr05 | 61384304 | 61384887 | unknown protein |  |
| Gorai.005G231400.1 | Chr05 | 61384764 | 61390164 | DNA/RNA polymerases superfamily protein |  |
| Gorai.005G231500.1 | Chr05 | 61391721 | 61394717 | Ribosomal RNA large subunit methyltransferase N |  |
| Gorai.005G231600.1 | Chr05 | 61395865 | 61400127 | Ribosomal protein L1p/L10e family |  |
| Gorai.005G231700.1 | Chr05 | 61401682 | 61402383 | unknown protein |  |
| Gorai.005G231800.1 | Chr05 | 61427381 | 61432101 | Vacuolar-sorting receptor |  |
| Gorai.005G231900.1 | Chr05 | 61433948 | 61435082 | Transcription factor bHLH84 |  |
| Gorai.005G232000.1 | Chr05 | 61462450 | 61468342 | RING/U-box superfamily protein |  |
| Gorai.005G232100.1 | Chr05 | 61471894 | 61475067 | Mitotic checkpoint protein BUB3 |  |
| Gorai.005G232200.1 | Chr05 | 61478922 | 61481160 | Pentatricopeptide repeat-containing protein At5g38730 |  |
| Gorai.005G232300.1 | Chr05 | 61484807 | 61486093 | Zinc finger protein ZAT10 |  |
| Gorai.005G232400.1 | Chr05 | 61498000 | 61501105 | BTB/POZ domain-containing protein NPY2 |  |
| Gorai.005G232500.1 | Chr05 | 61507927 | 61509072 | LOB domain-containing protein 38 |  |
| Gorai.005G232600.1 | Chr05 | 61513664 | 61517515 | Family of unknown function (DUF566) |  |
| Gorai.005G232700.1 | Chr05 | 61523538 | 61524652 | Ribosomal protein S14p/S29e family protein |  |
| Gorai.005G232800.1 | Chr05 | 61525429 | 61527595 | Regulator of Vps4 activity in the MVB pathway protein |  |
| Gorai.005G232900.1 | Chr05 | 61527596 | 61529668 | Uncharacterized protein At2g23090 |  |
| Gorai.005G233000.1 | Chr05 | 61538410 | 61541232 | Probable protein phosphatase 2C |  |
| Gorai.005G233100.1 | Chr05 | 61543421 | 61546433 | unknown protein |  |
| Gorai.005G233200.1 | Chr05 | 61546434 | 61547804 | NAD(P)-binding Rossmann-fold superfamily protein |  |
| Gorai.005G233300.1 | Chr05 | 61550697 | 61554250 | Pentatricopeptide repeat-containing protein At5g02860 |  |
| Gorai.005G233400.1 | Chr05 | 61556733 | 61558567 | SWIB/MDM2 domain superfamily protein |  |
| Gorai.005G233500.1 | Chr05 | 61568090 | 61569848 | arabinogalactan protein 18 | NAU2190 (HDC_c14_31.3) |
| Gorai.005G233600.1 | Chr05 | 61572415 | 61574820 | Protein of unknown function (DUF620) |  |
| Gorai.005G233700.1 | Chr05 | 61593261 | 61603281 | transducin family protein / WD-40 repeat family protein |  |
| Gorai.005G233800.1 | Chr05 | 61605124 | 61605816 | Gibberellin-regulated family protein |  |
| Gorai.005G233900.1 | Chr05 | 61607532 | 61608956 | Thioredoxin domain-containing protein 9 homolog |  |
| Gorai.005G234000.1 | Chr05 | 61611085 | 61614210 | pumilio 12 |  |
| Gorai.005G234100.1 | Chr05 | 61614949 | 61619006 | unknown protein |  |
| Gorai.005G234200.1 | Chr05 | 61624195 | 61626605 | Probable indole-3-acetic acid-amido synthetase GH3.1 |  |
| Gorai.005G234300.1 | Chr05 | 61650694 | 61655882 | Homeodomain-like protein with RING/FYVE/PHD-type zinc finger domain |  |
| Gorai.005G234400.1 | Chr05 | 61657737 | 61659491 | unknown protein |  |
| Gorai.005G234500.1 | Chr05 | 61660659 | 61662257 | unknown protein |  |
| Gorai.005G234600.1 | Chr05 | 61675857 | 61683506 | Limonoid UDP-glucosyltransferase |  |
| Gorai.005G234700.1 | Chr05 | 61709890 | 61711287 | UDP-glycosyltransferase 84B1 |  |
| Gorai.005G234800.1 | Chr05 | 61722000 | 61724123 | Probable cinnamyl alcohol dehydrogenase |  |
| Gorai.005G234900.1 | Chr05 | 61741646 | 61742938 | Transcription factor MYB44 |  |
| Gorai.005G235000.1 | Chr05 | 61748897 | 61750406 | unknown protein |  |
| Gorai.005G235100.1 | Chr05 | 61751810 | 61753488 | Reticulon family protein |  |
| Gorai.005G235200.1 | Chr05 | 61752925 | 61755574 | Protein TIC 21, chloroplastic |  |
| Gorai.005G235300.1 | Chr05 | 61756106 | 61760186 | sodium:hydrogen antiporter 1 |  |
| Gorai.005G235400.1 | Chr05 | 61760893 | 61761909 | stress enhanced protein 1 |  |
| Gorai.005G235500.1 | Chr05 | 61761910 | 61763650 | unknown protein |  |
| Gorai.005G235600.1 | Chr05 | 61770909 | 61772957 | CYCLIN D3;1 |  |
| Gorai.005G235700.1 | Chr05 | 61788313 | 61791360 | Calcium-dependent lipid-binding (CaLB domain) family protein |  |
| Gorai.005G235800.1 | Chr05 | 61791898 | 61796769 | PTEN 2 |  |
| Gorai.005G235900.1 | Chr05 | 61798099 | 61800954 | Granulin repeat cysteine protease family protein |  |
| Gorai.005G236000.1 | Chr05 | 61802819 | 61803136 | RNA polymerase subunit beta |  |
| Gorai.005G236100.1 | Chr05 | 61806100 | 61807778 | Uncharacterized protein At4g06744 |  |
| Gorai.005G236200.1 | Chr05 | 61819341 | 61820906 | Uncharacterized protein At4g06744 |  |
| Gorai.005G236300.1 | Chr05 | 61827133 | 61828554 | Protein of unknown function (DUF579) |  |
| Gorai.005G236400.1 | Chr05 | 61834591 | 61837827 | Cystathionine beta-synthase (CBS) family protein |  |
| Gorai.005G236500.1 | Chr05 | 61842262 | 61846388 | Polyadenylate-binding protein |  |
| Gorai.005G236600.1 | Chr05 | 61847520 | 61853751 | E3 ubiquitin-protein ligase MARCH6 |  |
| Gorai.005G236700.1 | Chr05 | 61855736 | 61860727 | Plant protein of unknown function (DUF869) |  |
| Gorai.005G236800.1 | Chr05 | 61860851 | 61863980 | O-acyltransferase WSD1 |  |
| Gorai.005G236900.1 | Chr05 | 61870257 | 61871219 | 40S ribosomal protein S16 |  |
| Gorai.005G237000.1 | Chr05 | 61871500 | 61874088 | O-acyltransferase WSD1 |  |
| Gorai.005G237100.1 | Chr05 | 61875516 | 61877238 | Caffeoyl-CoA O-methyltransferase |  |
| Gorai.005G237200.1 | Chr05 | 61883498 | 61885062 | U-box domain-containing protein |  |
| Gorai.005G237300.1 | Chr05 | 61893154 | 61895584 | Thioredoxin superfamily protein |  |
| Gorai.005G237400.1 | Chr05 | 61898814 | 61901502 | ABSCISIC ACID-INSENSITIVE 5-like protein |  |
| Gorai.005G237500.1 | Chr05 | 61905789 | 61909650 | Pentatricopeptide repeat-containing protein At4g33990 |  |
| Gorai.005G237600.1 | Chr05 | 61914099 | 61915078 | Protein of unknown function (DUF1685) |  |
| Gorai.005G237700.1 | Chr05 | 61920843 | 61922026 | unknown protein |  |
| Gorai.005G237800.1 | Chr05 | 61952148 | 61956240 | Abscisic acid 8'-hydroxylase |  |
| Gorai.005G237900.1 | Chr05 | 61962363 | 61962602 | Pyruvate kinase family protein |  |
| Gorai.005G238000.1 | Chr05 | 61970240 | 61971909 | redox responsive transcription factor 1 |  |
| Gorai.005G238100.1 | Chr05 | 61972723 | 61975006 | Vacuolar import/degradation, Vid27-related protein |  |
| Gorai.005G238200.1 | Chr05 | 61975167 | 61976845 | unknown protein |  |
| Gorai.005G238300.1 | Chr05 | 61977772 | 61982819 | Leucine-rich repeat (LRR) family protein |  |
| Gorai.005G238400.1 | Chr05 | 61987261 | 61988062 | unknown protein |  |
| Gorai.005G238500.1 | Chr05 | 61989026 | 61999674 | autophagy 2 |  |
| Gorai.005G238600.1 | Chr05 | 62004116 | 62007294 | AP2/B3-like transcriptional factor family protein |  |
| Gorai.005G238700.1 | Chr05 | 62008255 | 62012047 | AP2/B3-like transcriptional factor family protein |  |
| Gorai.005G238800.1 | Chr05 | 62024681 | 62029309 | Protein of unknown function DUF829, transmembrane 53 |  |
| Gorai.005G238900.1 | Chr05 | 62034529 | 62036115 | Cyclin-dependent kinase inhibitor family protein |  |
| Gorai.005G239000.1 | Chr05 | 62046353 | 62048869 | Probable carboxylesterase |  |
| Gorai.005G239100.1 | Chr05 | 62050758 | 62051720 | Probable carboxylesterase |  |
| Gorai.005G239200.1 | Chr05 | 62058528 | 62059472 | Probable carboxylesterase |  |
| Gorai.005G239300.1 | Chr05 | 62060616 | 62065473 | Acyltransferase-like protein At1g54570, chloroplastic |  |
| Gorai.005G239400.1 | Chr05 | 62070333 | 62071799 | Probable carboxylesterase |  |
| Gorai.005G239500.1 | Chr05 | 62080730 | 62082565 | receptor-like kinase 1 |  |
| Gorai.005G239600.1 | Chr05 | 62086149 | 62086993 | unknown protein |  |
| Gorai.005G239700.1 | Chr05 | 62101442 | 62117043 | phragmoplast orienting kinesin 2 |  |
| Gorai.005G239800.1 | Chr05 | 62117268 | 62121647 | Serine/threonine-protein kinase SRK2E |  |
| Gorai.005G239900.1 | Chr05 | 62128132 | 62133763 | SWIB complex BAF60b domain-containing protein |  |
| Gorai.005G240000.1 | Chr05 | 62138381 | 62141548 | RNA-binding protein |  |
| Gorai.005G240100.1 | Chr05 | 62144166 | 62144749 | unknown protein |  |
| Gorai.005G240200.1 | Chr05 | 62149701 | 62153270 | Peptidyl-prolyl cis-trans isomerase CYP40 |  |
| Gorai.005G240300.1 | Chr05 | 62154834 | 62157145 | unknown protein |  |
| Gorai.005G240400.1 | Chr05 | 62165103 | 62170159 | CDPK-related protein kinase |  |
| Gorai.005G240500.1 | Chr05 | 62171479 | 62173086 | Tetratricopeptide repeat (TPR)-like superfamily protein |  |
| Gorai.005G240600.1 | Chr05 | 62178149 | 62181631 | C2H2 and C2HC zinc fingers superfamily protein |  |
| Gorai.005G240700.1 | Chr05 | 62183428 | 62184740 | Protein of unknown function (DUF1645) |  |
| Gorai.005G240800.1 | Chr05 | 62188045 | 62191607 | Polyadenylate-binding protein RBP47B |  |
| Gorai.005G240900.1 | Chr05 | 62206571 | 62209880 | B3 domain-containing transcription factor VRN1 |  |
| Gorai.005G241000.1 | Chr05 | 62217125 | 62217490 | unknown protein |  |
| Gorai.005G241100.1 | Chr05 | 62219918 | 62220280 | unknown protein |  |
| Gorai.005G241200.1 | Chr05 | 62226424 | 62228247 | VP1/ABI3-like 3 |  |
| Gorai.005G241300.1 | Chr05 | 62233359 | 62233760 | AP2/B3-like transcriptional factor family protein |  |
| Gorai.005G241400.1 | Chr05 | 62241282 | 62244473 | Ubiquitin-like domain-containing CTD phosphatase |  |
| Gorai.005G241500.1 | Chr05 | 62245288 | 62247389 | ribosomal protein L30 family protein | Unig28F05 (HDC_c14_27.6), close to BNL3259 |
| Gorai.005G241600.1 | Chr05 | 62248314 | 62253342 | Basic-leucine zipper (bZIP) transcription factor family protein |  |
| Gorai.005G241700.1 | Chr05 | 62260116 | 62264395 | MLO-like protein |  |
| Gorai.005G241800.1 | Chr05 | 62265956 | 62266988 | Ferredoxin-1, chloroplastic |  |
| Gorai.005G241900.1 | Chr05 | 62272225 | 62274964 | Ferredoxin-1, chloroplastic |  |
| Gorai.005G242000.1 | Chr05 | 62276369 | 62282849 | T-complex protein 11 |  |
| Gorai.005G242100.1 | Chr05 | 62295487 | 62301414 | serine hydroxymethyltransferase 6 |  |
| Gorai.005G242200.1 | Chr05 | 62299657 | 62308096 | Potassium channel AKT2/3 |  |
| Gorai.005G242300.1 | Chr05 | 62316676 | 62318622 | Probable receptor-like protein kinase At1g11050 |  |
| Gorai.005G242400.1 | Chr05 | 62322134 | 62326117 | Glycine cleavage T-protein family |  |
| Gorai.005G242500.1 | Chr05 | 62338956 | 62345118 | Pentatricopeptide repeat-containing protein At1g10910, chloroplastic |  |
| Gorai.005G242600.1 | Chr05 | 62347429 | 62353334 | SWI/SNF complex subunit SWI3D |  |
| Gorai.005G242700.1 | Chr05 | 62355218 | 62356069 | Probable CCR4-associated factor 1 homolog |  |
| Gorai.005G242800.1 | Chr05 | 62358759 | 62361455 | Ras-related protein Rab7 |  |
| Gorai.005G242900.1 | Chr05 | 62364171 | 62370154 | Coatomer subunit gamma |  |
| Gorai.005G243000.1 | Chr05 | 62370632 | 62374762 | Guanine nucleotide-binding protein subunit beta-2 |  |
| Gorai.005G243100.1 | Chr05 | 62379642 | 62385251 | Glucan endo-1,3-beta-glucosidase |  |
| Gorai.005G243200.1 | Chr05 | 62392157 | 62396805 | Adenylyl cyclase-associated protein |  |
| Gorai.005G243300.1 | Chr05 | 62398990 | 62403180 | Probable receptor-like serine/threonine-protein kinase At4g34500 |  |
| Gorai.005G243400.1 | Chr05 | 62413319 | 62414170 | Probable CCR4-associated factor 1 homolog |  |
| Gorai.005G243500.1 | Chr05 | 62414783 | 62415326 | unknown protein |  |
| Gorai.005G243600.1 | Chr05 | 62416104 | 62417134 | unknown protein |  |
| Gorai.005G243700.1 | Chr05 | 62417671 | 62420301 | NADH dehydrogenase [ubiquinone] iron-sulfur protein 8-B, mitochondrial |  |
| Gorai.005G243800.1 | Chr05 | 62421676 | 62425720 | Probable LRR receptor-like serine/threonine-protein kinase At2g16250 |  |
| Gorai.005G243900.1 | Chr05 | 62432126 | 62433175 | cold, circadian rhythm, and rna binding 2 |  |
| Gorai.005G244000.1 | Chr05 | 62441761 | 62442796 | RAD-like 1 |  |
| Gorai.005G244100.1 | Chr05 | 62447795 | 62450259 | 3-ketoacyl-CoA synthase |  |
| Gorai.005G244200.1 | Chr05 | 62461406 | 62463671 | basic helix-loop-helix (bHLH) DNA-binding superfamily protein |  |
| Gorai.005G244300.1 | Chr05 | 62466754 | 62469651 | Putative pectinesterase |  |
| Gorai.005G244400.1 | Chr05 | 62477068 | 62479105 | Aureusidin synthase |  |
| Gorai.005G244500.1 | Chr05 | 62497981 | 62500560 | Isoflavone reductase homolog P3 |  |
