# Supplementary material for: Inheritance and QTL Mapping of Leaf Nutrient Concentration in a Cotton Inter-Specific Derived RIL Population
Source: PLoS One. 2015 May 28;10(5):e0128100. doi: 10.1371/journal.pone.0128100 (PMC4447399; doi:10.1371/journal.pone.0128100)
Supplement: S3 Table — (DOCX) [file pone.0128100.s004.docx]

**S3 Table. Pearson correlation coefficients of five macronutrient, Na concentrations and K/Na ratio in leaves with yield components and fibre properties in the RIL population based on a combined data analysis over two seasons.**

| Trait | | P | | K | | | Ca | | Mg | | S | Na | K/Na ratio |
| --- | --- | --- | --- | --- | --- | --- | --- | --- | --- | --- | --- | --- | --- |
| **Yield components** | |  | |  | | |  | |  | |  |  |  |
| Lint % | | -0.19 | | -0.06 | | | 0.25* | | 0.35** | | 0.21 | 0.22 | -0.28* |
| Boll weight | | -0.19 | | -0.29* | | | 0.32** | | 0.41*** | | 0.34** | 0.44*** | -0.30* |
| Seed weight | | 0.03 | | 0.09 | | | -0.14 | | -0.09 | | -0.08 | 0.03 | 0.00 |
| Seed no./boll | | -0.18 | | -0.34** | | | 0.33** | | 0.40*** | | 0.35** | 0.42*** | -0.26* |
| Lint weight/seed | | -0.18 | | -0.01 | | | 0.17 | | 0.30* | | 0.16 | 0.25* | -0.28* |
| Fibre no/seed | | -0.08 | | -0.09 | | | 0.16 | | 0.09 | | 0.02 | 0.14 | -0.24* |
| **Fibre properties** |  | |  | |  | | |  | |  |  |  |  |
| Length | | -0.08 | | 0.05 | | -0.17 | | | -0.16 | | -0.07 | -0.20 | 0.14 |
| Uniformity | | -0.15 | | 0.02 | | 0.09 | | | 0.13 | | 0.25* | 0.15 | 0.05 |
| Short fibre index | | 0.11 | | 0.00 | | 0.04 | | | -0.04 | | -0.19 | -0.05 | -0.09 |
| Strength | | -0.01 | | 0.06 | | -0.16 | | | -0.21 | | -0.18 | -0.10 | 0.14 |
| Elongation | | -0.10 | | 0.02 | | -0.24* | | | -0.15 | | -0.15 | -0.15 | 0.05 |
| Micronaire | | -0.16 | | 0.00 | | 0.04 | | | 0.23 | | 0.13 | 0.23* | -0.10 |
| Maturity ratio | | -0.31** | | -0.03 | | 0.08 | | | 0.20 | | 0.18 | 0.31** | -0.12 |
| Fineness | | -0.10 | | 0.01 | | 0.06 | | | 0.27* | | 0.13 | 0.19 | -0.13 |
| Perimeter | | 0.17 | | 0.05 | | 0.02 | | | 0.23 | | 0.02 | 0.01 | -0.08 |

*, **, *** indicate significance at P≤0.05, 0.01, 0.001, respectively. n=68.
