# Supplementary material for: Inheritance and QTL Mapping of Leaf Nutrient Concentration in a Cotton Inter-Specific Derived RIL Population
Source: PLoS One. 2015 May 28;10(5):e0128100. doi: 10.1371/journal.pone.0128100 (PMC4447399; doi:10.1371/journal.pone.0128100)
Supplement: S4 Table — (DOCX) [file pone.0128100.s005.docx]

**S4 Table. Pearson correlation coefficients of five micronutrient concentrations in leaves with yield components and fibre properties for the RIL population based on a combined data analysis over two seasons.**

| Trait | | Fe | | Mn | | B | | Cu | Zn |
| --- | --- | --- | --- | --- | --- | --- | --- | --- | --- |
| **Yield components** | |  | |  | |  | |  |  |
| Lint% | | 0.18 | | 0.24* | | -0.03 | | -0.04 | -0.10 |
| Boll weight | | 0.01 | | 0.31* | | 0.05 | | 0.09 | -0.10 |
| Seed weight | | -0.03 | | 0.05 | | 0.08 | | 0.24 | -0.04 |
| Seed no./boll | | -0.01 | | 0.27* | | 0.02 | | 0.04 | -0.05 |
| Lint weight/seed | | 0.14 | | 0.29* | | 0.00 | | 0.08 | -0.15 |
| Fibre no/seed | | 0.26* | | 0.16 | | 0.02 | | 0.02 | -0.09 |
| **Fibre properties** |  | |  | |  | |  |  |  |
| Length | | -0.02 | | -0.05 | | -0.10 | | -0.04 | -0.14 |
| Uniformity | | -0.12 | | 0.12 | | -0.04 | | 0.07 | -0.08 |
| Short fibre index | | 0.23* | | -0.01 | | 0.05 | | 0.02 | 0.15 |
| Strength | | -0.01 | | -0.06 | | -0.07 | | -0.04 | -0.09 |
| Elongation | | -0.41*** | | -0.30* | | 0.01 | | -0.27* | -0.28* |
| Micronaire | | -0.11 | | 0.11 | | 0.00 | | -0.02 | -0.03 |
| Maturity ratio | | -0.10 | | 0.09 | | 0.01 | | -0.04 | -0.09 |
| Fineness | | -0.14 | | 0.15 | | 0.05 | | 0.02 | -0.06 |
| Perimeter | | -0.10 | | 0.13 | | 0.05 | | 0.06 | 0.01 |

*, **, *** indicate significance at P≤0.05, 0.01, 0.001, respectively. n=68.
